# Supplementary material for: Multiplexed volumetric CLEM enabled by scFvs provides insights into the cytology of cerebellar cortex
Source: Nat Commun. 2024 Aug 5;15:6648. doi: 10.1038/s41467-024-50411-z (PMC11300613; doi:10.1038/s41467-024-50411-z)
Supplement: Supplementary file 1 — Reporting Summary [file 41467_2024_50411_MOESM1_ESM.pdf]

nature portfolio

Corresponding author(s): Xiaomeng Han, Xiaotang Lu, Jeff Lichtman

Last updated by author(s): Jun 12, 2024

Reporting Summary

Nature Portfolio wishes to improve the reproducibility of the work that we publish. This form provides structure for consistency and transparency in reporting. For further information on Nature Portfolio policies, see our Editorial Policies and the Editorial Policy Checklist.

Statistics

For all statistical analyses, confirm that the following items are present in the figure legend, table legend, main text, or Methods section.

n/a	Confirmed	
☐	☒	The exact sample size (*n*) for each experimental group/condition, given as a discrete number and unit of measurement
☐	☒	A statement on whether measurements were taken from distinct samples or whether the same sample was measured repeatedly
☐	☒	The statistical test(s) used AND whether they are one- or two-sided<br>*Only common tests should be described solely by name; describe more complex techniques in the Methods section.*
☐	☒	A description of all covariates tested
☒	☐	A description of any assumptions or corrections, such as tests of normality and adjustment for multiple comparisons
☐	☒	A full description of the statistical parameters including central tendency (e.g. means) or other basic estimates (e.g. regression coefficient) AND variation (e.g. standard deviation) or associated estimates of uncertainty (e.g. confidence intervals)
☐	☒	For null hypothesis testing, the test statistic (e.g. *F*, *t*, *r*) with confidence intervals, effect sizes, degrees of freedom and *P* value noted<br>*Give P values as exact values whenever suitable.*
☒	☐	For Bayesian analysis, information on the choice of priors and Markov chain Monte Carlo settings
☒	☐	For hierarchical and complex designs, identification of the appropriate level for tests and full reporting of outcomes
☒	☐	Estimates of effect sizes (e.g. Cohen's *d*, Pearson's *r*), indicating how they were calculated

Our web collection on statistics for biologists contains articles on many of the points above.

Software and code

Policy information about availability of computer code

Data collection	Zeiss Zen Black (2.3 SP1), Zeiss Zen Blue (3.10), Zeiss Zen Pro (3.10) software was used for confocal fluorescence microscopy image collection. Zeiss Atlas 5 software was used for single-beam electron microscopy image collection. Zeiss ZEN multiSEM software (3.10) was used for multi-beam electron microscopy image collection.
Data analysis	Zeiss Zen Blue software (3.10), Zeiss Zen Pro (3.10), FIJI/ImageJ, and the FIJI/ImageJ plugin BigWarp were used for confocal fluorescence microscopy image processing and fluorescence image/electron microscopy image co-registration. mb_aligner (https://github.com/Gilhirith/mb_aligner) was used for multi-beam electron microscopy image stitching and alignment. Flood-Filling Networks (https://github.com/google/ffn) and mEMbrain (https://github.com/emmay78/mEMbrain) were used for multi-beam electron microscopy image stack segmentation. VAST (https://lichtman.rc.fas.harvard.edu/vast/) and Neuroglancer (https://github.com/google/neuroglancer) were used for volumetric correlated light and electron microscopy dataset visualization, 3D reconstruction, and analysis. PyTorch code (https://connectomics.readthedocs.io/en/latest/tutorials/mito.html) was used for mitochondria segmentation. Cellpose (https://github.com/mouseland/cellpose) was used for synaptic vesicle detection. Prism-GraphPad (10.2.1) was used for statistical analysis.

For manuscripts utilizing custom algorithms or software that are central to the research but not yet described in published literature, software must be made available to editors and reviewers. We strongly encourage code deposition in a community repository (e.g. GitHub). See the Nature Portfolio guidelines for submitting code & software for further information.

Data

Policy information about availability of data

All manuscripts must include a data availability statement. This statement should provide the following information, where applicable:

- Accession codes, unique identifiers, or web links for publicly available datasets
- A description of any restrictions on data availability
- For clinical datasets or third party data, please ensure that the statement adheres to our policy

The DNA sequences of the scFvs genereated in this study have been deposited in GenBank under accession codes PP840912-PP840919. The expression plasmids of the scFvs generated in this study have been deposited at Addgene: https://www.addgene.org/browse/article/28238227/;.The vCLEM dataset and its segmentation are publicly available through the Neuroglancer link in the source data file. The authors declare that all the representative data supporting the findings of this study are available within the paper and its supplementary information files. Source data are provided with this paper. Other data that support the findings of this study are available from the corresponding authors upon request.

Research involving human participants, their data, or biological material

Policy information about studies with human participants or human data. See also policy information about sex, gender (identity/presentation), and sexual orientation and race, ethnicity and racism.

Reporting on sex and gender	n/a
Reporting on race, ethnicity, or other socially relevant groupings	n/a
Population characteristics	n/a
Recruitment	n/a
Ethics oversight	n/a

Note that full information on the approval of the study protocol must also be provided in the manuscript.

Field-specific reporting

Please select the one below that is the best fit for your research. If you are not sure, read the appropriate sections before making your selection.

☒ Life sciences ☐ Behavioural & social sciences ☐ Ecological, evolutionary & environmental sciences

For a reference copy of the document with all sections, see nature.com/documents/nr-reporting-summary-flat.pdf

Life sciences study design

All studies must disclose on these points even when the disclosure is negative.

Sample size	During the development of the new technique described in this study, at least 200 samples were used. Three samples were used for the three replicates of the multi-color vCLEM experiments. The volumes chosen were to test the applicability of this method to connectomic datasets.
Data exclusions	During the development of the new technique, fluorescence microscopy images that were out-of-focus, or from sections that had debris and tears were excluded; electron microscopy images that were out-of-focus, had inappropriate brightness/contrast setting, or had debris, tears, or wrinkles were excluded.<br><br>For the vCLEM experiment that generated the vCLEM dataset, only electron microscopy images that had severe tears were excluded. These exclusion criteria are conventional and comparable to other electron microscopy studies.<br><br>For the analysis of the mossy fiber terminals, mossy fiber arborizations that were en-passant but not terminal were excluded.
Replication	All proof-of-principle scFv immunolabeling experiment was replicated 2-3 times, and was successful over the course of the study. Multi-color vCLEM experiment enabled by scFv immunolabeling was replicated 3 times. We only collected serial section electron microscopy data from one of the experiments. A sample number of 10 for each category was chosen for the mossy fiber terminal analysis, as it is sufficient for running a t-test. .
Randomization	There was no randomization because there were no experimental or control groups.
Blinding	In the mossy fiber terminal analysis, quantifications were automatically carried out by computer algorithms.

Reporting for specific materials, systems and methods

We require information from authors about some types of materials, experimental systems and methods used in many studies. Here, indicate whether each material, system or method listed is relevant to your study. If you are not sure if a list item applies to your research, read the appropriate section before selecting a response.

Materials & experimental systems

n/a	Involved in the study
☐	☒ Antibodies
☐	☒ Eukaryotic cell lines
☒	☐ Palaeontology and archaeology
☐	☒ Animals and other organisms
☒	☐ Clinical data
☐	☒ Dual use research of concern
☒	☐ Plants

Methods

n/a	Involved in the study
☒	☐ ChIP-seq
☒	☐ Flow cytometry
☒	☐ MRI-based neuroimaging

Antibodies

Antibodies used

Anti-GFP nanobody (Enh), Alexa Fluor 647 (final conc.: 0.01 mg/ml); Anti-GFP scFv (N86/38), 5-TAMRA (final conc.: 0.01 mg/ml); Anti-CALB1/calbindin scFv (L109/57), 5-TAMRA or Alexa Fluor 488 (final conc.: 0.01 mg/ml); Anti-GFAP-R416WT scFv (N206B/9), 5-TAMRA (final conc.: 0.01 mg/ml); Anti-VGlut1 scFv (N28/9), 5-TAMRA or Alexa Fluor 532 (final conc.: 0.015 mg/ml); Anti-PSD-95 scFv (K28/43), 5-TAMRA (final conc.: 0.01 mg/ml); Anti-Kv1.2 scFv (K14/16), Alexa Fluor 594 (final conc.: 0.02 mg/ml); Anti-Parvalbumin scFv (L114/81), Alexa Fluor 647 (final conc.: 0.01 mg/ml); Anti-Neuropeptide Y Antibody (L115/13), Alexa Fluor 594 (final conc.: 0.01 mg/ml).

GFP Polyclonal Antibody, Alexa Fluor 647 (1:200, ThermoFisher, A-31852); Anti-CALB1/calbindin Antibody, (L109/57) (1:200, Antibodies Incorporated, 75-448); Anti-CALB1/calbindin Antibody, (L109/30) (22.5 µg/ml, NeuroMab); Anti-CALB1/calbindin Antibody, (L109/57) (3 µg/ml, NeuroMab); Anti-CALB1/calbindin Polyclonal Antibody, (1:100, Synaptic System, 214 005); Anti-GFAP-R416WT Antibody (N206B/9) (1:200, Antibodies Incorporated, 75-279); Anti-GFAP Antibody (N206B/8) (11.1 µg/ml, NeuroMab); Anti-GFAP Antibody (N206B/9) (1:5, NeuroMab); Anti-VGlut1 Antibody (N28/9) (1:200, Antibodies Incorporated, 75-066); Anti-VGlut2 Antibody (N29/29) (1:200, Antibodies Incorporated, 75-067); Anti-PSD-95 Antibody (K28/43) (1:200, Antibodies Incorporated, 75-028); Anti-Kv1.2 K+ Channel Antibody (K14/16) (1:500, Antibodies Incorporated, 75-008); Anti-Kv2.1 K+ Channel Antibody (In house polyclonal rabbit antibody, Trimmer 1991) (1:20); Anti-Parvalbumin Antibody (L114/81) (1:200, Antibodies Incorporated, 75-479); Anti-Parvalbumin Antibody (L114/81 R, IgG2a) (1:2, NeuroMab); Anti-Parvalbumin Polyclonal Antibody (1:250, Abcam, ab11427); Anti-FLAG Polyclonal Antibody (2.5 µg/ml, MIllipore, F7425); Anti-Neuropeptide Y Antibody (L115/13) (1:200, Antibodies Incorporated, 75-456); Anti-Pan-QKI Antibody (N147/6) (2.5 µg/ml, NeuroMab); Anti-GAD1 Antibody (L127/8) (5 µg/ml, NeuroMab);

Goat anti-Mouse IgG1 Cross-Adsorbed Secondary Antibody, Alexa Fluor 488 (1:100, ThermoFisher, A-21121); Goat anti-Mouse IgG2a Cross-Adsorbed Secondary Antibody, Alexa Fluor 488 (1:100 or 2 µg/ml, ThermoFisher, A-21131); Goat anti-Mouse IgG2b Cross-Adsorbed Secondary Antibody, Alexa Fluor 488 (1:100 or 2 µg/ml, ThermoFisher, A-21141); AffiniPure™ Goat Anti-Guinea Pig IgG, $F(ab')_2$ fragment specific, Alexa Fluor 594 (1:100, Jackson Immuno Research, 106-585-006); AffiniPure™ Goat Anti-Rabbit IgG, $F(ab')_2$ fragment specific, Alexa Fluor 594 (1:100, Jackson Immuno Research, 111-585-006); Goat anti-Mouse IgG1 Secondary Antibody, CF770 (1:2500, Biotium, 20254-1); Donkey anti-Rabbit IgG (H+L) Secondary Antibody, CF750 (2 µg/ml, Biotium, 20298-1); Goat anti-Mouse IgG2b Secondary Antibody, Alexa Fluor 594 (1:2500, ThermoFisher, A-21145); Goat anti-Rabbit IgG (H+L) Secondary Antibody, Alexa Fluor 488 (1:2500, ThermoFisher, A-11008); Goat anti-Rabbit IgG (H+L) Secondary Antibody, Alexa Fluor 647 (2 µg/ml, ThermoFisher, A-21244); Goat anti-Mouse IgG1 Secondary Antibody, Alexa Fluor 555 (2 µg/ml, ThermoFisher, A-21127); Goat anti-Mouse IgG2b Secondary Antibody, Alexa Fluor 555 (2 µg/ml, ThermoFisher, A-21147); Goat anti-Mouse IgG1 Secondary Antibody, Alexa Fluor 647 (2 µg/ml, ThermoFisher, A-21240); Goat anti-Mouse IgG2b Secondary Antibody, Alexa Fluor 647 (2 µg/ml, ThermoFisher, A-21242)

Validation

Anti-GFP nanobody (Enh), Alexa Fluor 647 was validated in this publication: https://www.nature.com/articles/s41592-018-0177-x; Anti-GFP scFv (N86/38), 5-TAMRA; Anti-CALB1/calbindin scFv (L109/57), 5-TAMRA or Alexa Fluor 488; Anti-GFAP-R416WT scFv (N206B/9), 5-TAMRA; Anti-VGlut1 scFv (N28/9), 5-TAMRA or Alexa Fluor 532;); Anti-PSD-95 scFv (K28/43), 5-TAMRA; Anti-Kv1.2 scFv (K14/16), Alexa Fluor 594; Anti-Parvalbumin scFv (L114/81), Alexa Fluor 647; Anti-Neuropeptide Y Antibody (L115/13), Alexa Fluor 594 were validated in this study.

GFP Polyclonal Antibody was validated with immunocytochemistry and Western blot by the manufacturer: https://www.thermofisher.com/antibody/product/GFP-Antibody-Polyclonal/A-31852;
Anti-CALB1/calbindin Antibody, (L109/57) was validated with immunohistochemistry and Western blot by the manufacturer: https://www.antibodiesinc.com/products/anti-calb1-calbindin-antibody-l109-57-75-448;
Anti-CALB1/calbindin Antibody, (L109/39) was validated with IF ICC, Brain Immuno blot and brain IHC by the manufacturer: https://neuromab.ucdavis.edu/datasheet/L109_39.pdf;
Anti-CALB1/calbindin Polyclonal Antibody, (Synaptic System, 214 005) was validated with Western blot, ICC-IF and IHC-IF by the manufacturer: https://www.sysy.com/product/214005;
Anti-GFAP-R416WT Antibody (N206B/9) was validated with immunohistochemistry and Western blot by the manufacturer: https://www.antibodiesinc.com/products/anti-gfap-r416wt-antibody-n206b-9-75-279;
Anti-GFAP Antibody, (N206A/8) was validated with IF ICC, Brain Immuno blot and brain IHC by the manufacturer: https://neuromab.ucdavis.edu/datasheet/N206A_8.pdf;
Anti-GFAP Antibody, (N206A/9) was validated with IF ICC, Brain Immuno blot and brain IHC by the manufacturer: /https://neuromab.ucdavis.edu/datasheet/N206B_9.pdf;
Anti-VGlut1 Antibody (N28/9) was validated with immunohistochemistry and Western blot by the manufacturer: https://www.antibodiesinc.com/products/anti-vglut1-antibody-n28-9-75-066;
Anti-VGlut2 Antibody (N29/29) was validated with immunohistochemistry and Western blot by the manufacturer: https://www.antibodiesinc.com/products/anti-vglut2-antibody-n29-29-75-067;
Anti-PSD-95 Antibody (K28/43) was validated with immunocytochemistry, immunohistochemistry and Western blot by the manufacturer: https://www.antibodiesinc.com/products/anti-psd-95-antibody-k28-43-75-028;
Anti-Kv1.2 K+ Channel Antibody (K14/16) was validated with immunohistochemistry and Western blot by the manufacturer: https://www.antibodiesinc.com/products/anti-kv1-2-k-channel-antibody-k14-16-75-008;
Anti-Kv2.1 In-house Polyclonal Antibody was validated in (Trimmer, PNAS, 1991, PMCID: PMC53011);
Anti-Parvalbumin Antibody (L114/81) was validated with array tomography, immunohistochemistry and Western blot by the manufacturer: https://www.antibodiesinc.com/products/anti-parvalbumin-antibody-l114-81-75-479;
Anti-Parvalbumin Antibody (L114/81 R) was validated in (Andrews et al., 2019, Elife);
Anti-Parvalbumin Polyclonal Antibody (Abcam, ab11427) was validated with ICC-IF and IHC-P by the manufacturer: https://www.abcam.com/products/primary-antibodies/parvalbumin-antibody-ab11427.html;
Neuropeptide Y Antibody (L115/13) was validated with array tomography and immunohistochemistry by the manufacturer: https://www.antibodiesinc.com/products/anti-neuropeptide-y-antibody-l115-13-75-456;
Anti-FLAG rabbit polyclonal antibody was validated with dot blot, immunoblotting, immunoprecipitation, and immunocytochemistry by the manufacturer: https://www.sigmaaldrich.com/US/en/product/sigma/f7425;
Pan-QKI Antibody (N147/6) was validated with immunoblot, immunocytochemistry and immunohistochemistry by the manufacturer: https://neuromab.ucdavis.edu/datasheet/N147_6.pdf;
Pan-GAD1 Antibody (L127/8) was validated with immunoblot, immunocytochemistry, immunohistochemistry, and array tomography by the manufacturer: hhttps://neuromab.ucdavis.edu/datasheet/L127_8.pdf;

Goat anti-Mouse IgG1 Cross-Adsorbed Secondary Antibody, Alexa Fluor 488 was validated with immunocytochemistry and immunohistochemistry by the manufacturer: https://www.thermofisher.com/antibody/product/Goat-anti-Mouse-IgG1-Cross-Adsorbed-Secondary-Antibody-Polyclonal/A-21121;
Goat anti-Mouse IgG2a Cross-Adsorbed Secondary Antibody, Alexa Fluor 488 was validated with immunocytochemistry by the manufacturer: https://www.thermofisher.com/antibody/product/Goat-anti-Mouse-IgG2a-Cross-Adsorbed-Secondary-Antibody-Polyclonal/A-21131;
Goat anti-Mouse IgG2b Cross-Adsorbed Secondary Antibody, Alexa Fluor 488 was validated with immunocytochemistry by the manufacturer: https://www.thermofisher.com/antibody/product/Goat-anti-Mouse-IgG2b-Cross-Adsorbed-Secondary-Antibody-Polyclonal/A-21141.
AffiniPure™ Goat Anti-Guinea Pig IgG, F(ab')$_2$ fragment specific, lexa Fluor 594 was validated with immunoelectrophoresis and/or ELISA by the manufacturer: https://www.jacksonimmuno.com/catalog/products/106-585-006.
AffiniPure™ Goat Anti-Rabbit IgG, F(ab')$_2$ fragment specific, Alexa Fluor 594 was validated with immunoelectrophoresis and/or ELISA by the manufacturer: https://www.jacksonimmuno.com/catalog/products/111-585-006.
Goat anti-Mouse IgG1 Secondary Antibody, CF770 was validated with Western blot, IF and IHC by the manufacturer: https://biotium.com/product/goat-anti-mouse-igg1/?attribute_pa_conjugation=cf770;
Donkey anti-Rabbit IgG (H+L) Secondary Antibody, CF750 was validated with Western blot, IF and IHC by the manufacturer: https://biotium.com/product/donkey-anti-rabbit-igg-hl-highly-cross-adsorbed-cf-dye-storm/;
Goat anti-Mouse IgG2b Secondary Antibody, Alexa Fluor 594 was validated with immunocytochemistry and immunohistochemistry by the manufacturer: https://www.thermofisher.com/antibody/product/Goat-anti-Mouse-IgG2b-Cross-Adsorbed-Secondary-Antibody-Polyclonal/A-21145;
Goat anti-Rabbit IgG (H+L) Secondary Antibody, Alexa Fluor 488 was validated with immunocytochemistry and immunohistochemistry by the manufacturer: https://www.thermofisher.com/antibody/product/Goat-anti-Rabbit-IgG-H-L-Cross-Adsorbed-Secondary-Antibody-Polyclonal/A-11008.
Goat anti-Rabbit IgG (H+L) Secondary Antibody, Alexa Fluor 647 was validated with immunocytochemistry and flow cytometry by the manufacturer: https://www.thermofisher.com/antibody/product/Goat-anti-Rabbit-IgG-H-L-Cross-Adsorbed-Secondary-Antibody-Polyclonal/A-21244;
Goat anti-Mouse IgG1 Secondary Antibody, Alexa Fluor 555 was validated with immunocytochemistry and immunohistochemistry by the manufacturer: https://www.thermofisher.com/antibody/product/Goat-anti-Mouse-IgG1-Cross-Adsorbed-Secondary-Antibody-Polyclonal/A-21127;
Goat anti-Mouse IgG2b Secondary Antibody, Alexa Fluor 555 was validated with Western blot, immunocytochemistry and immunohistochemistry by the manufacturer: https://www.thermofisher.com/antibody/product/Goat-anti-Mouse-IgG2b-Cross-Adsorbed-Secondary-Antibody-Polyclonal/A-21147;
Goat anti-Mouse IgG1 Secondary Antibody, Alexa Fluor 647 was validated with Western blot, immunocytochemistry and immunohistochemistry by the manufacturer: https://www.thermofisher.com/antibody/product/Goat-anti-Mouse-IgG1-Cross-Adsorbed-Secondary-Antibody-Polyclonal/A-21240;
Goat anti-Mouse IgG2b Secondary Antibody, Alexa Fluor 647 was validated with Western blot, immunocytochemistry and immunohistochemistry by the manufacturer: https://www.thermofisher.com/antibody/product/Goat-anti-Mouse-IgG2b-Cross-Adsorbed-Secondary-Antibody-Polyclonal/A-21242;

Eukaryotic cell lines

Policy information about cell lines and Sex and Gender in Research

Cell line source(s)	Expi293 cells were purchased from ThermoFisher; COS-1 cells (ATCC cat# CRL-1650); HEK293T cells (ATCC cat# CRL-3216)
Authentication	Didn't perform authentication.
Mycoplasma contamination	Didn't perform test for mycoplasma contamination.
Commonly misidentified lines (See ICLAC register)	No commonly misidentified cell lines were used in the study.

Animals and other research organisms

Policy information about studies involving animals; ARRIVE guidelines recommended for reporting animal research, and Sex and Gender in Research

Laboratory animals	Animals used in the study were adult (8-12 weeks) YFP-H mice (Jackson Laboratory, https://www.jax.org/strain/003782), adult (8-12 weeks) C58BL/6J mice (Jackson Laboratory, https://www.jax.org/strain/000664), adult female Sprague Dawley rats. The vCLEM dataset was collected from a female adult (8-12 weeks) C57BL/6J mouse. All experiments using mice were conducted according to US National Institutes of Health guidelines and approved by the Institutional Animal Care and Use Committee at Harvard University (protocol number 24-08-4). The housing conditions are: Dark/light cycle: Lights on at 6 am and off at 8 pm (14L:10D); Ambient temperature: 22 °C ± 1; Humidity: 30-70%. All experiments for rat immunohistochemistry were performed in strict compliance with the University of California Davis ethical regulations for studies involving animals as approved by the University of California Davis Animal Care and Use Committee (protocol #: 23734). UC Davis Animal Welfare Assurance Number (Vertebrate Animals) A-3433-01. The housing conditions are: Dark/light cycle: Lights on at 7 am and off at 7 pm (12L:12D); Ambient temperature: 20-26 °C; Humidity: 30-70%.
Wild animals	n/a
Reporting on sex	Sex was not considered in this study because this is a technology development study. All animals used were females.
Field-collected samples	n/a
Ethics oversight	All experiments involving animals in this study were conducted according to US National Institutes of Health guidelines and approved by the Committee on Animal Care at Harvard University and UC Davis.

Note that full information on the approval of the study protocol must also be provided in the manuscript.
