# Supplementary material for: Multiplexed volumetric CLEM enabled by scFvs provides insights into the cytology of cerebellar cortex
Source: Nat Commun. 2024 Aug 5;15:6648. doi: 10.1038/s41467-024-50411-z (PMC11300613; doi:10.1038/s41467-024-50411-z)
Supplement: Supplementary file 3 — Supplementary information [file 41467_2024_50411_MOESM3_ESM.pdf]

Multiplexed volumetric CLEM enabled by scFvs provides new insights into the cytology of cerebellar cortex

Xiaomeng Han[1*], Xiaotang Lu[1*], Peter H. Li[2], Shuohong Wang[1], Richard Schalek[1], Yaron Meirovitch[1], Zudi Lin[3], Jason Adhinarta[4], Karl D. Murray[5], Leah M. MacNiven[5], Daniel R. Berger[1], Yuelong Wu[1], Tao Fang[6], Elif Sevde Meral[7], Shadnan Asraf[8], Hidde Ploegh[6], Hanspeter Pfister[5], Donglai Wei[4], Viren Jain[2], James S. Trimmer[5], Jeff W. Lichtman[1*]

[1] Department of Molecular and Cellular Biology, Harvard University, Cambridge, MA
[2] Google Research, Mountain View, CA
[3] School of Engineering and Applied Sciences, Harvard University, Cambridge, MA
[4] Computer Science Department, Boston College, Chestnut Hill, MA
[5] Department of Physiology and Membrane Biology, University of California Davis School of Medicine, Davis, CA
[6] Program of Cellular and Molecular Medicine, Boston Children’s Hospital, Boston, MA
[7] Bezmialem Vakif University School of Medicine, Istanbul, Turkey
[8] School of Public Health, University of Massachusetts Amherst, Amherst, MA

[*] Corresponding Authors: xiaomenghan@fas.harvard.edu, xiaotang_lu@fas.harvard.edu, jeff@mcb.harvard.edu

Supplementary Materials

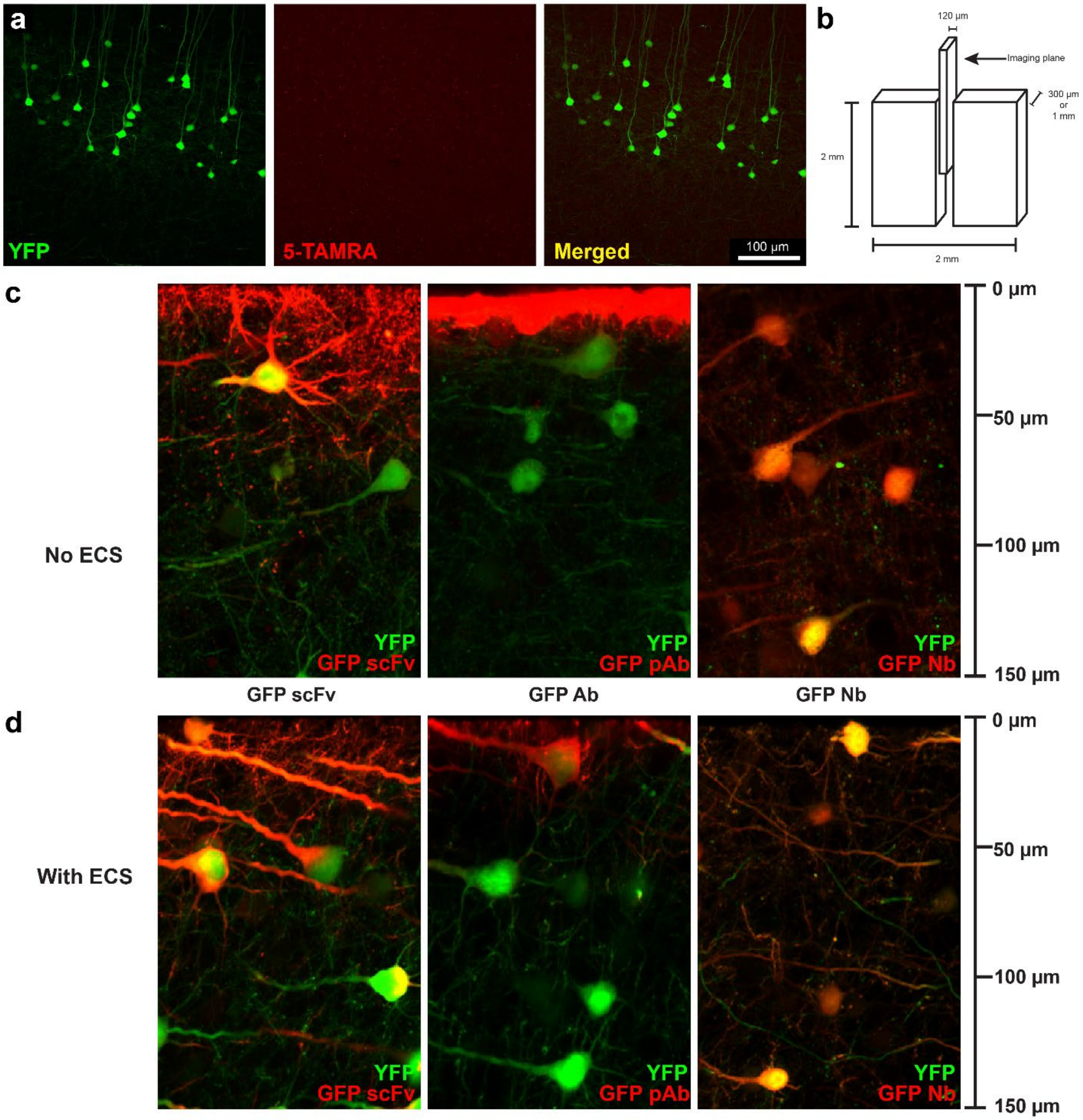

Sup. Figure 1. Characterization of the GFP-specific scFv.

a, Representative confocal images (n=3 experiments, all experiments mentioned refer to independent experiments) from the cerebral cortex of an unlabeled YFP-H mouse showing no signal in the red channel. **b**, Schematic drawing showing how the tissue penetration was evaluated. After a brain tissue section of 2 mm x 2 mm x 300 µm (or 1 mm) was immunolabeled, 120-µm sections across the thickness (300 µm or 1 mm) were cut and collected. The 120-µm section from the middle was imaged in a way as the arrow indicates. **c** and **d**, Tissue penetration depth comparison of scFvs, pAbs, and nanobodies without or with the preservation of ECS (n=2 experiments). Without ECS preservation, after a free-floating incubation of seven days, the scFv penetrated to a depth of ~60 µm into the tissue; the polyclonal anti-GFP antibody penetrated to a depth of ~10 µm; the nanobody can penetrate to a depth of ~150 µm. With ECS preservation, after a free-floating incubation of seven days, the scFv penetrated to a depth of >100 µm into the tissue; the polyclonal anti-GFP antibody penetrated to a depth of ~30 µm; the nanobody can

penetrate to a depth of ~150 µm.

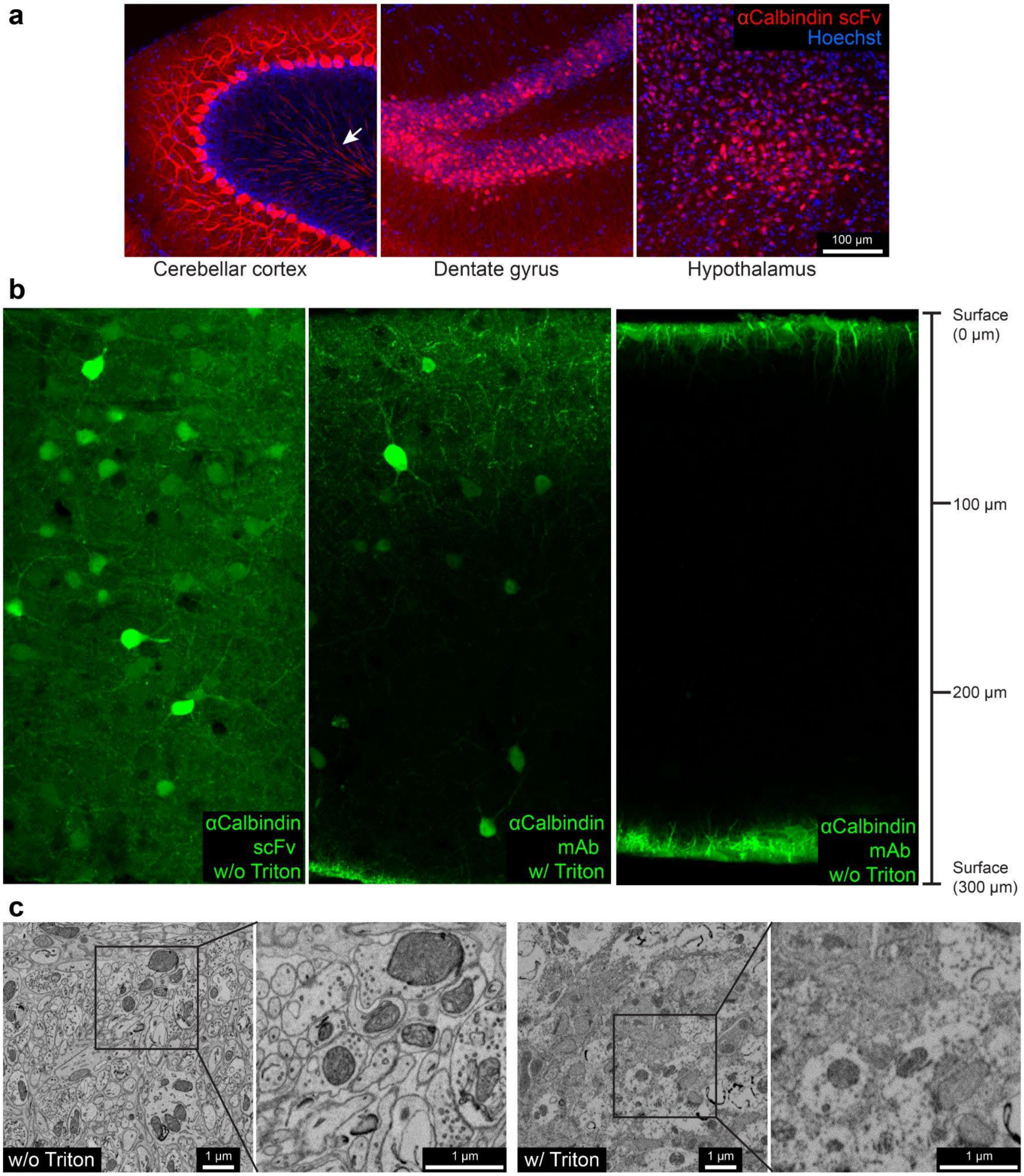

Sup. Figure 2. Immunolabeling results of anti-calbindin scFv and tissue penetration comparison.

a, Additional brain regions labeled with a calbindin-specific scFv probe conjugated with 5-TAMRA (n=3 experiments). The arrow in the left panel shows myelinated Purkinje cell axons in the granule layer. **b**, Tissue penetration depth comparison of scFvs, mAbs (plus secondary antibodies), and the role of detergents in improving the depth of labeling (n=2 experiments). **c**, Comparison of ultrastructure with and

without 0.5% Triton X-100 on scFv labeled samples (n=2 experiments). Boxed insets are shown at higher magnification in adjacent panels.

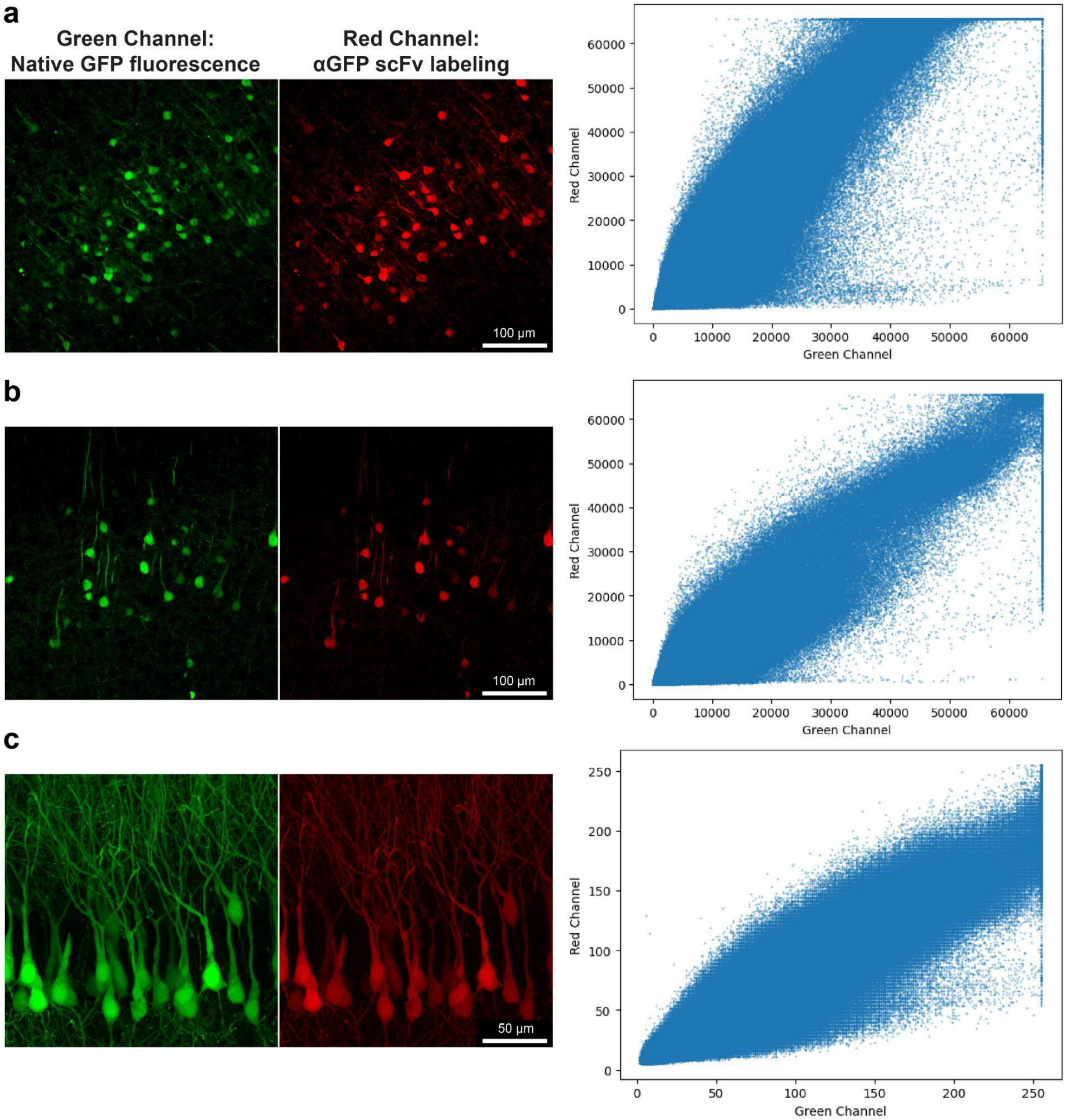

Sup. Figure 3. Pixel correlation scatter plots compare the native YFP fluorescence signal and the red fluorescence from the labeling of the GFP-specific scFv.

Cerebral cortex samples (**a** and **b**) and hippocampus (**c**) from YFP-H mice were immunolabeled with a GFP-specific scFv conjugated with the red fluorophore 5-TAMRA. The images are raw data without any brightness/contrast adjustment. **a** is the raw image data of Figure 1 b.

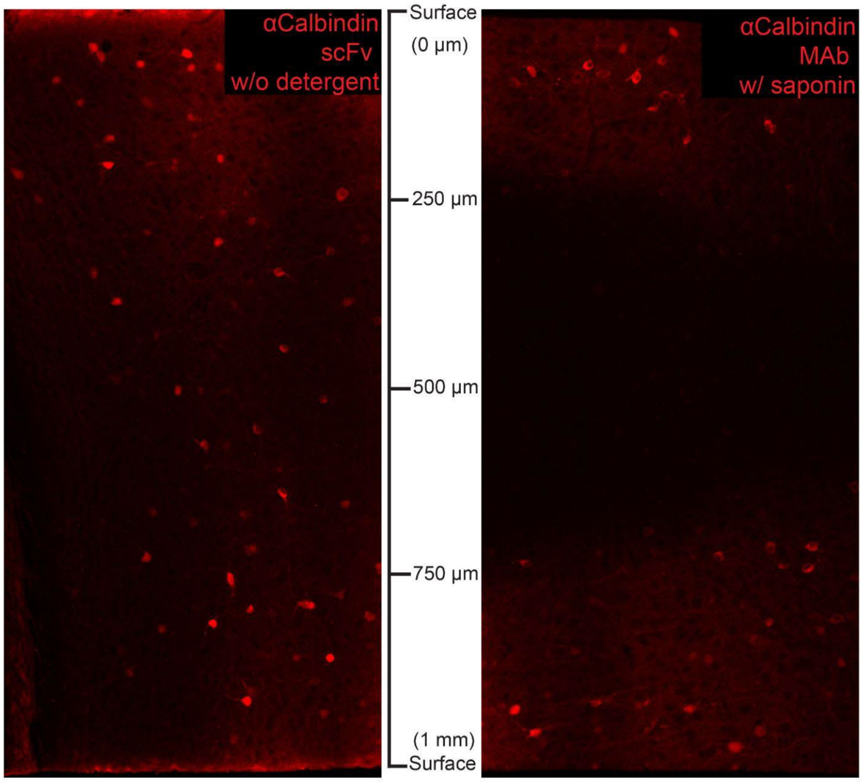

Sup. Figure 4. Penetration of the anti-calbindin scFv into a 1-mm tissue sample.

Tissue penetration depth comparison (n=2 experiments) of a calbindin-specific scFv without detergent and its parental mAb directly conjugated with fluorophores with 0.05% saponin on 1-mm cerebral cortex tissue sections with a 7-day incubation.

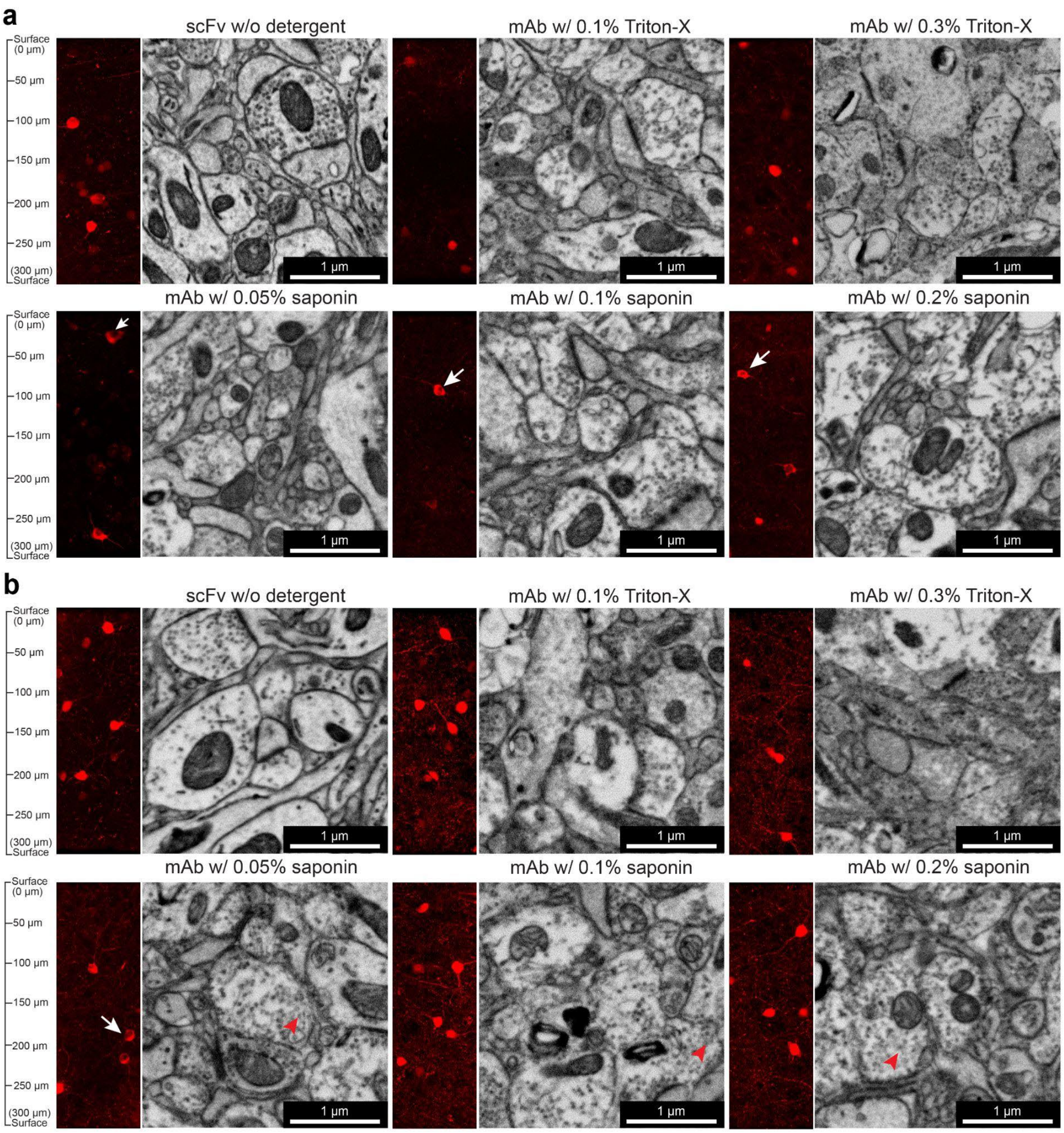

Sup. Figure 5. Tissue penetration depth comparison of scFvs in the absence of detergent and fluorophore-conjugated mAbs with the treatments of various concentrations of different detergents.

300-μm cerebral cortex sections (n=2 experiments in each category) were immunolabeled for one day (**a**) or seven days (**b**) with a calbindin-specific scFv conjugated with 5-TAMRA in the absence of detergent or with the scFv's parental mAb conjugated with FL550 in the presence of 0.1%, 0.3% Triton-X, or 0.05%, 0,1%, 0.2% saponin. Arrows indicate unlabeled cell nuclei. Arrowheads indicate granular textures associated with the treatment of saponin. EM was performed n=1 sample in each category.

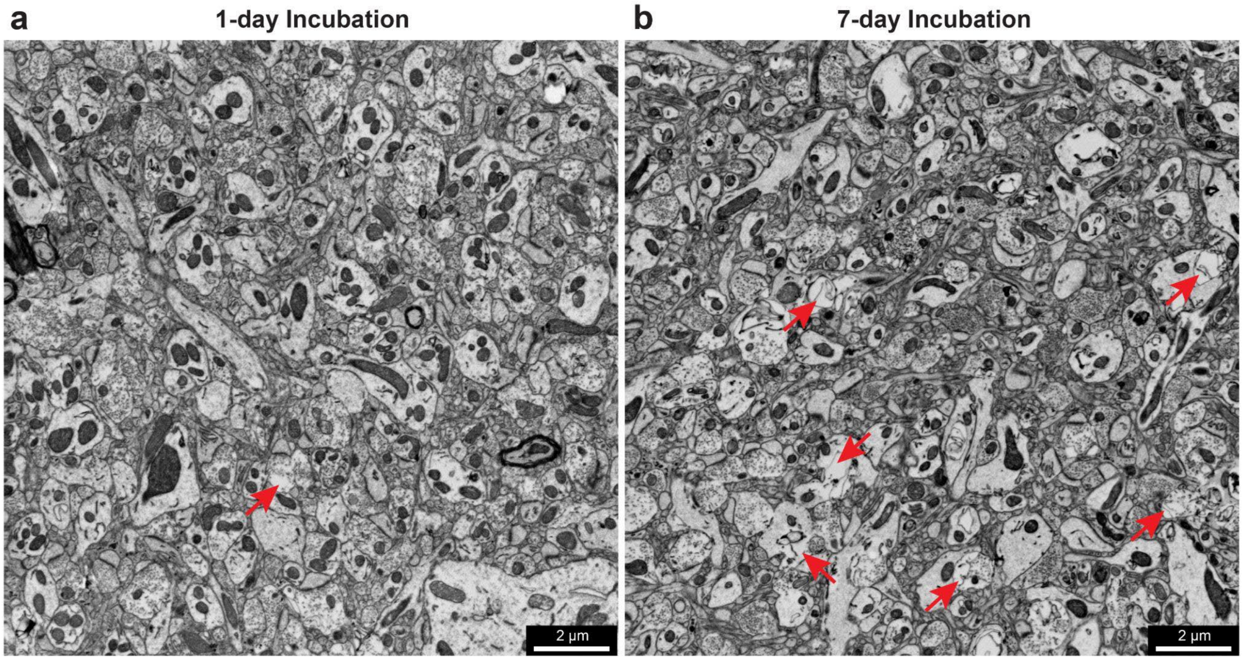

Sup. Figure 6. Ultrastructure comparison between samples incubated for one day or seven days (n=2 experiments).

Ultrastructure of locations close to the surfaces of 300-µm cerebral cortex sections immunolabeled for one day (**a**) or seven days (**b**). Arrows indicate artifacts.

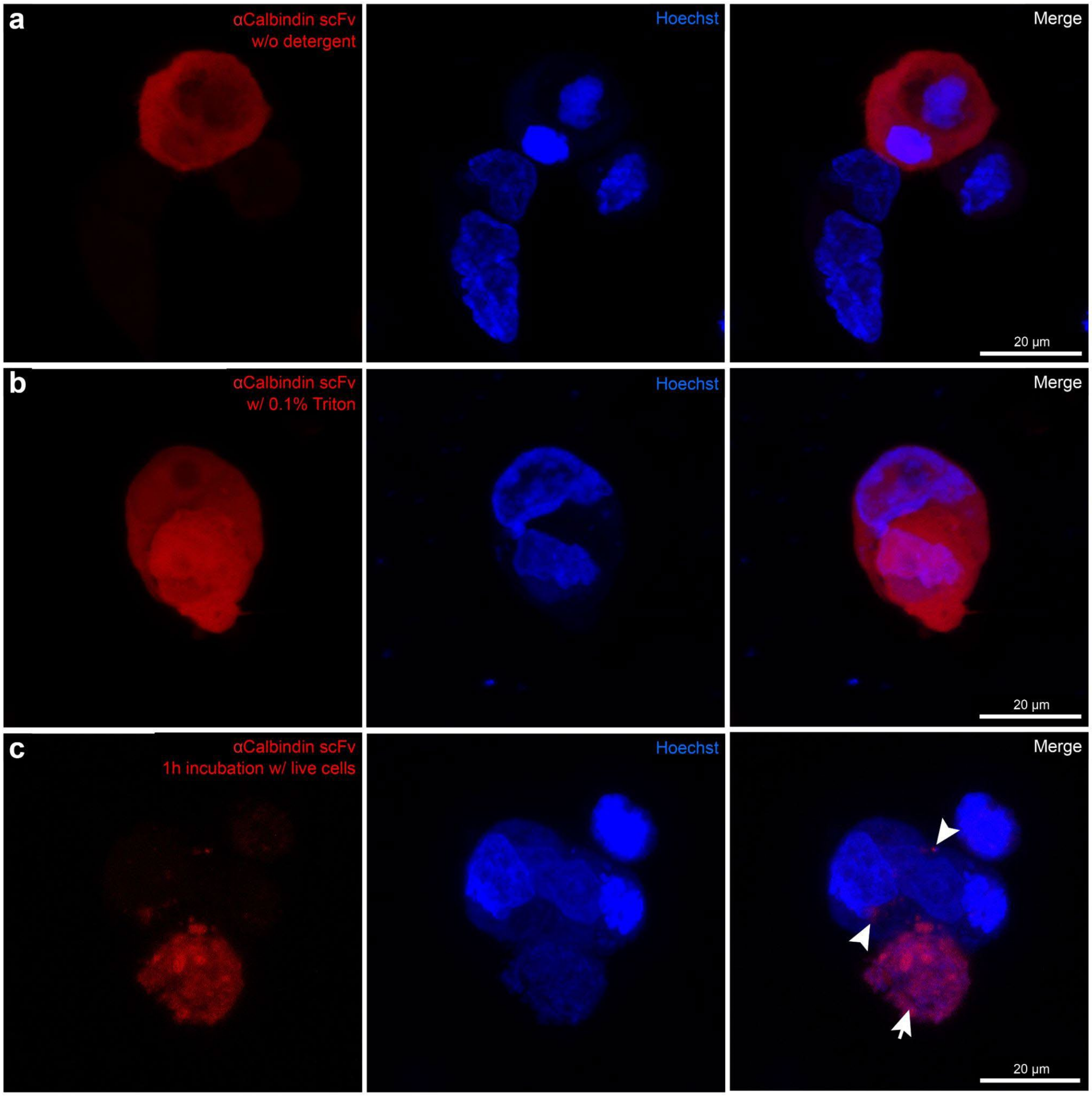

Sup. Figure 7. Penetration of the anti-calbindin scFv into fixed HEK cells or live cells (n=3 experiments).

Immunofluorescence immunocytochemistry on transiently transfected cells. HEK cells were transfected with a plasmid encoding Flag-tagged human calbindin. **a**, Chemically fixed cells were labeled overnight with Alexa594 anti-calbindin L109/57 scFv in the absence of detergent. **b**, Chemically fixed cells were labeled overnight with Alexa594 anti-calbindin L109/57 scFv with 0.1% Triton-X. **c**, Live cells were labeled with Alexa594 anti-calbindin L109/57 scFv. The arrow indicates the cell that has intracellular scFv labeling. Arrowheads indicate puncta labeling in other cells.

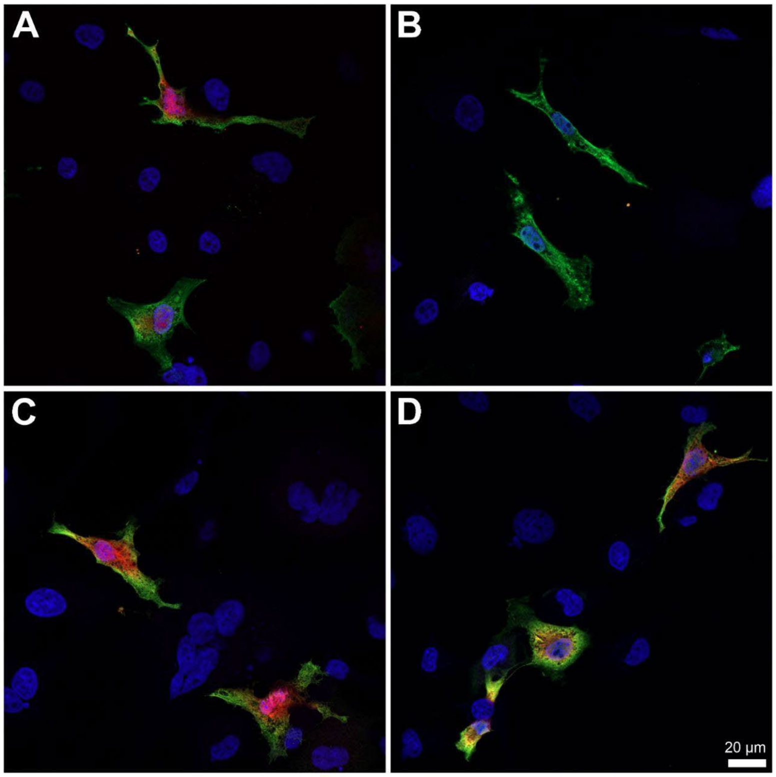

Sup. Figure 8. Penetration of the anti-calbindin scFv into fixed COS-1 cells (n=3 experiments).

Immunofluorescence immunocytochemistry on transiently transfected cells. COS-1 cells were transfected with a plasmid encoding Flag-tagged human calbindin. Cells in panels A and B were labeled for 1 hour after fixation and prior to detergent permeabilization with (**A**) Alexa594 anti-calbindin L109/57 scFv or (**B**) anti-calbindin mouse mAb L109/39 (scFv and mAb labeling in red). After permeabilization, cells were labeled with rabbit anti-Flag (green) to detect calbindin, and Hoechst nuclear dye (blue). For cells in panels C and D all immunolabeling was performed after fixation and detergent permeabilization with (**C**) Alexa594 anti-calbindin L109/57 scFv or (**D**) anti-calbindin mouse mAb L109/39 (scFv and mAb labeling in red). Cells were simultaneously labeled with rabbit anti-Flag (green), and Hoechst (blue). Cells in all panels were imaged at the same exposure.

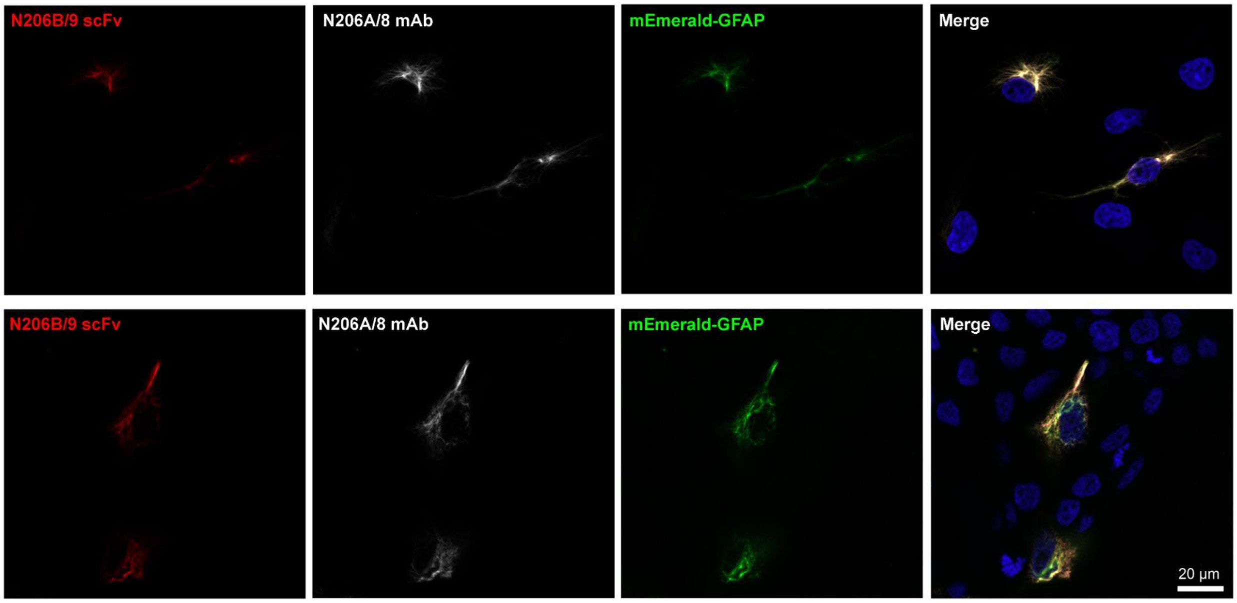

Sup. Figure 9. Validation of the anti-GFAP scFv with immunofluorescence immunocytochemistry (n=3 experiments).

Immunofluorescence immunocytochemistry on transiently transfected cells. COS-1 cells (top row) and HEK293T cells (bottom row). Cells were transfected with a plasmid encoding mEmerald-tagged human GFAP (green) and double immunolabeled with the 5-TAMRA-labeled anti-GFAP N206B/9 scFv (red) and the anti-GFAP N206A/8 mouse IgG1 mAb (white). Hoechst nuclear labeling is shown in blue.

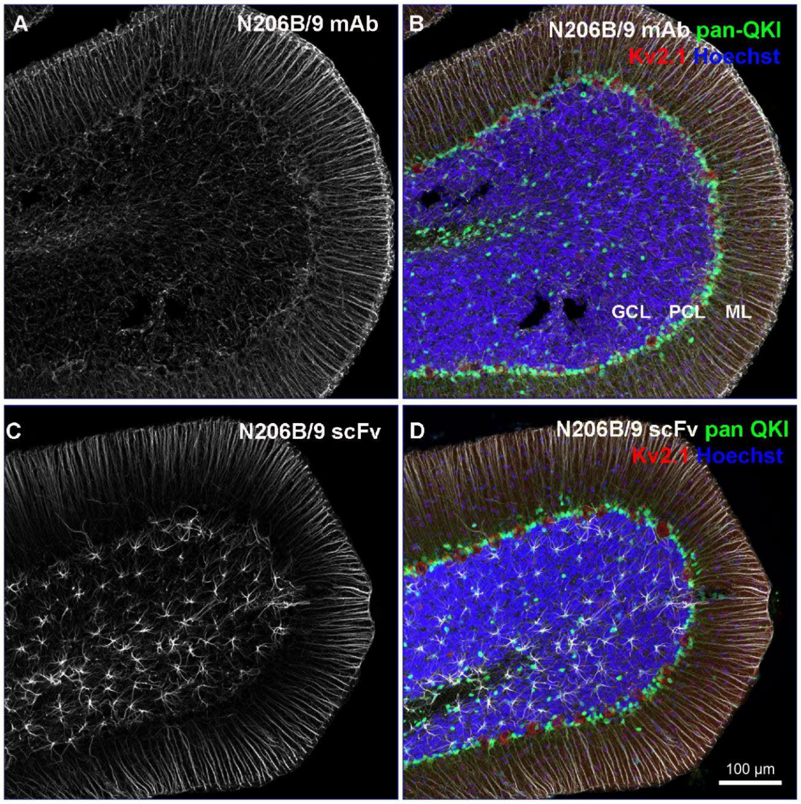

Sup. Figure 10. Validation of the anti-GFAP scFv with immunofluorescence immunohistochemistry (cerebellum) (n=3 experiments).

GFAP scFv and the original monoclonal antibody from which it was derived display the same tissue labeling pattern of a sagittal section through the rat cerebellum. **A**) Glial cells throughout the cerebellar granule cell layer (GCL) and prominent Bergmann glial process in the molecular layer (ML) are labeled with hybridoma-derived monoclonal antibody N206B/9. **B**) merged image includes labeling with a polyclonal rabbit antibody[1] against the neuronal potassium channel Kv2.1, a monoclonal antibody targeting glial-specific RNA binding protein QKI (N147/6), and nuclear-specific Hoechst labeling. **C**) An adjacent section labeled with N206B/9 derived scFv shows the same pattern of labeling. **D**) merged image with the same additional labeling as B.

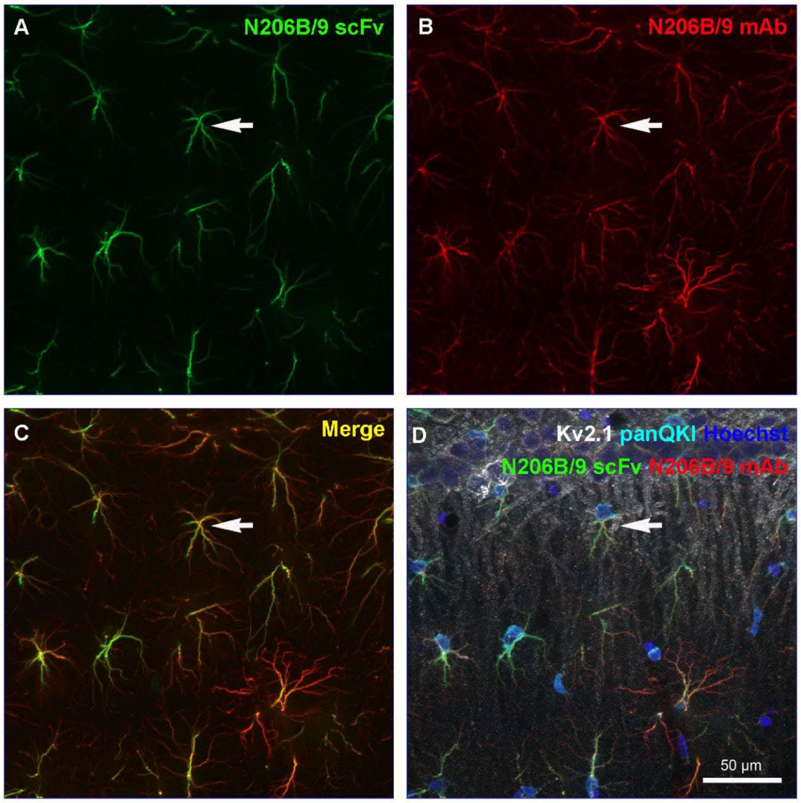

Sup. Figure 11. Validation of the anti-GFAP scFv with immunofluorescence immunohistochemistry (hippocampus) (n=3 experiments).

Validation of scFv labeling pattern against the hybridoma-generated monoclonal antibody N206B/9 from which it was derived. Multiplex immunofluorescent labeling of a sagittal section through rat hippocampal region CA1. **A**) 5-TAMRA conjugated N206B/9 derived scFv, **B**) Hybridoma derived monoclonal antibody N206B/9 indirectly labeled with Alexa fluor 647 conjugated goat anti-mouse IgG1 secondary antibody, **C**) merged images from A and B illustrating co-labeled astroglial cells (e.g., arrowheads). **D**) The same multiplex image shown in C with additional labeling for the neuronal-specific potassium channel Kv2.1, the glial-specific pan-QKI RNA binding protein, and the DNA marker Hoechst 33342.

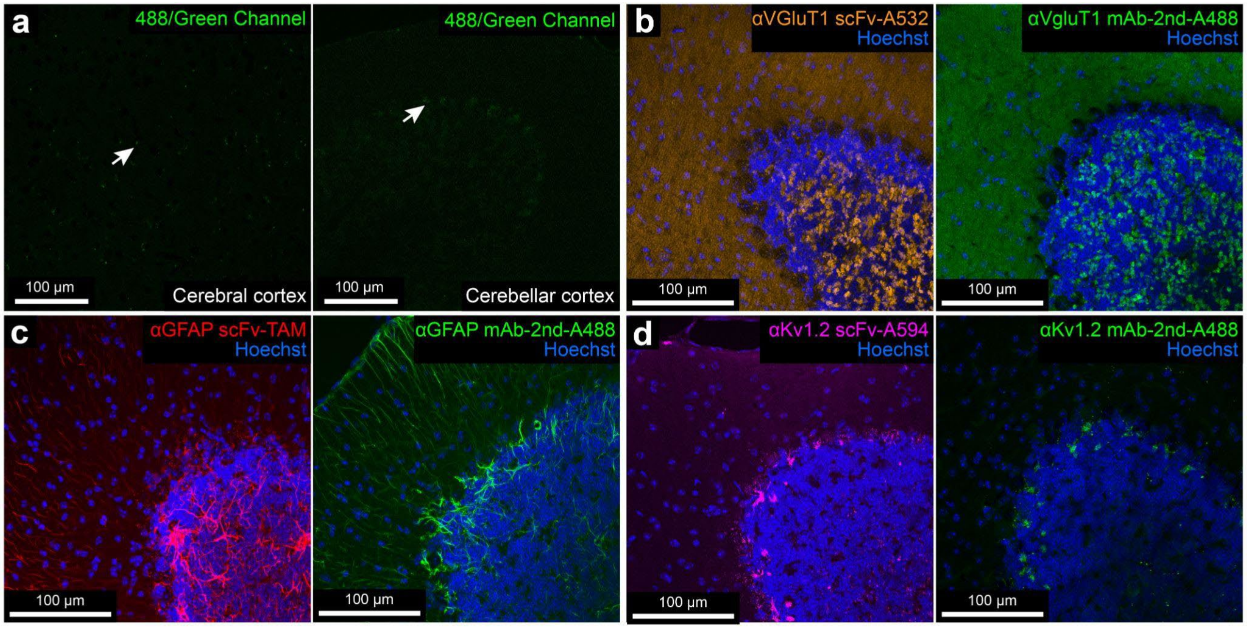

Sup. Figure 12. Validation by immunofluorescence immunohistochemistry of scFv probes and their parental mAbs (part 1).

a, Representative confocal images (n=3 experiments) from unlabeled cerebral cortex and cerebellar cortex of a wild-type mouse showed limited background in the 488/green channel. Arrows indicate background signals from lipofuscin granules. **b**-**d**, Cerebellum Crus 1 sections (n=3 experiments for each category) were immunolabeled with scFvs targeting VGluT1, GFAP, and Kv1.2; or these scFvs' parental mAbs and secondary antibodies conjugated with Alexa Fluor 488.

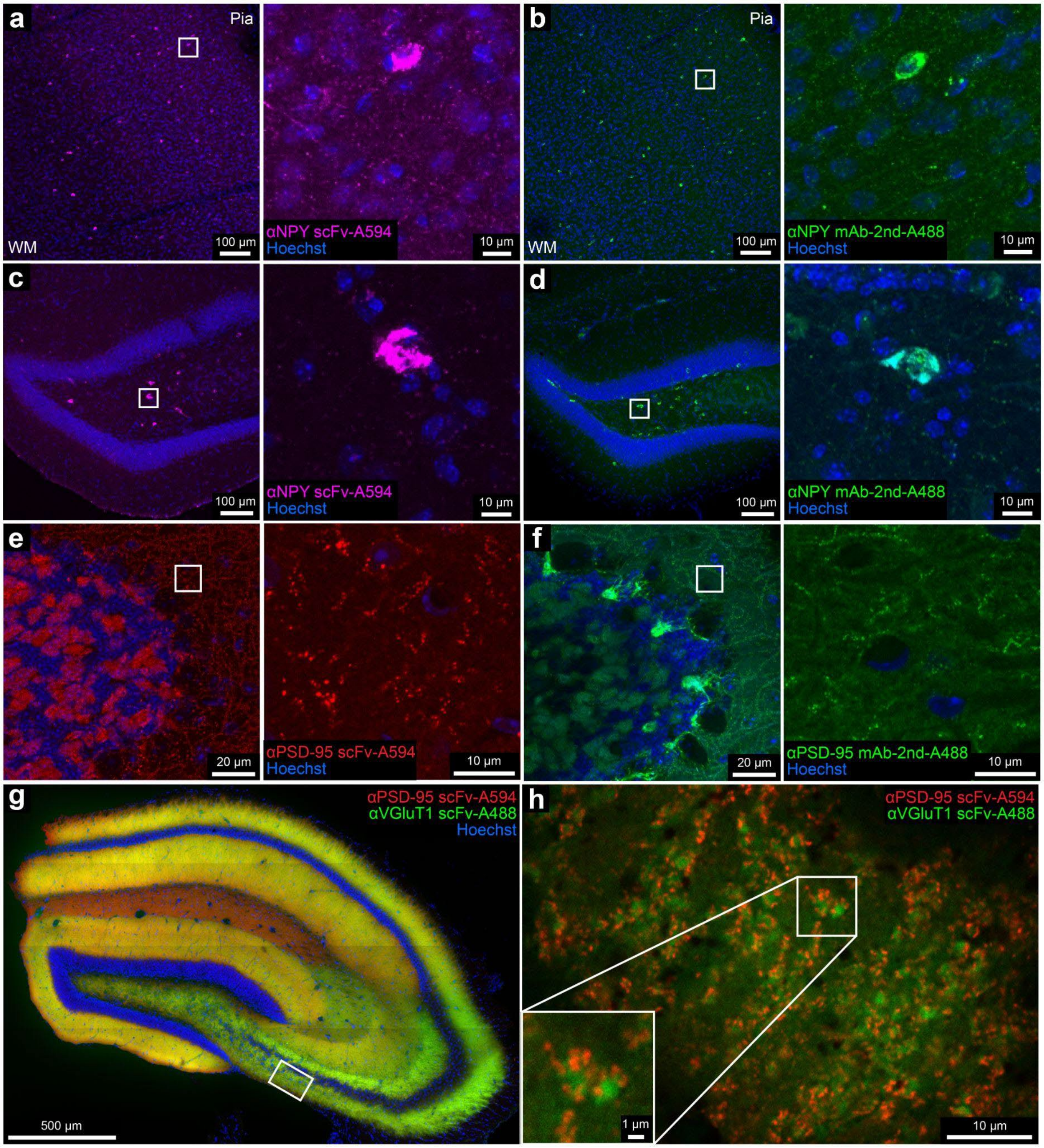

Sup. Figure 13. Validation by immunofluorescence immunohistochemistry of scFv probes and their parental mAbs (part 2).

a-**f**, Representative confocal images (n=2 experiments for each category) of different sections from the cerebral cortex (**a** and **b**) , the hippocampus (**c** and **d**), or the cerebellum Crus 1 (**e** and **f**) stained with scFvs targeting NPY or PSD-95; or their parental mAbs and secondary antibodies conjugated with Alexa Fluor 488. The right panels in **a**-**f** are enlarged boxed insets from the left panels. **g** and **h**, Representative confocal images of sections (n=2 experiments) from hippocampus stained with scFvs targeting PSD-95 or VGluT1. **h** is the boxed inset from **g** imaged at higher magnification. These experiments were performed

on samples prepared with ECS-preserving fixation protocol.

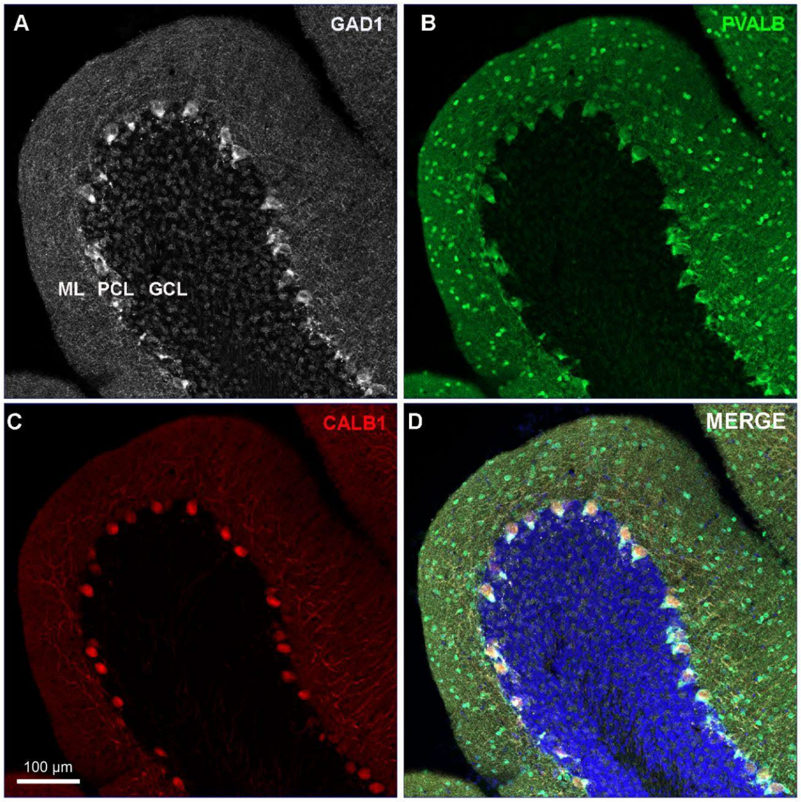

Sup. Figure 14. Validation of the anti-calbindin and anti-parvalbumin scFvs' parental mAbs with Immunofluorescence immunohistochemistry. (n=3 experiments).

Labeling pattern of original mAbs used to generate scFvs against parvalbumin and calbindin in rat cerebellum. Sagittal section through cerebellum labeled with monoclonal antibodies L127/8 (**A**, GAD1), L114/8 R (**B**, PARV, parvalbumin), and L109/57 (**C**, CALB1, calbindin). The merged image (**D**) shows the colocalized pattern of labeling within the Purkinje cell layer (PCL). ML, molecular layer, GCL, granule cell layer.

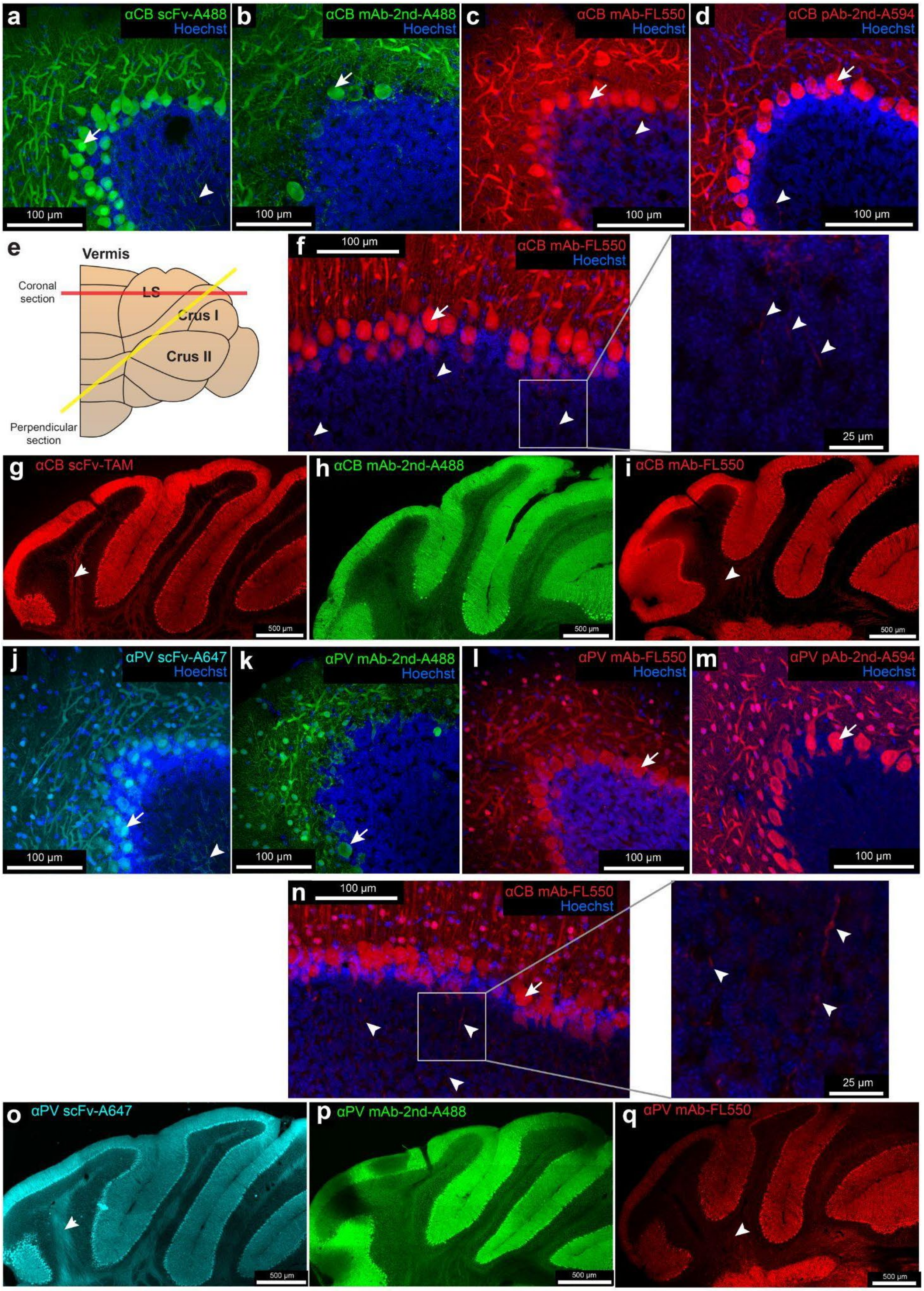
a
αCB scFv-A488
Hoechst
100 µm
b
αCB mAb-2nd-A488
Hoechst
100 µm
c
αCB mAb-FL550
Hoechst
100 µm
d
αCB pAb-2nd-A594
Hoechst
100 µm
e
Vermis
Coronal section
LS
Crus I
Crus II
Perpendicular section
f
100 µm
αCB mAb-FL550
Hoechst
25 µm
g
αCB scFv-TAM
500 µm
h
αCB mAb-2nd-A488
500 µm
i
αCB mAb-FL550
500 µm
j
αPV scFv-A647
Hoechst
100 µm
k
αPV mAb-2nd-A488
Hoechst
100 µm
l
αPV mAb-FL550
Hoechst
100 µm
m
αPV pAb-2nd-A594
Hoechst
100 µm
n
100 µm
αCB mAb-FL550
Hoechst
25 µm
o
αPV scFv-A647
500 µm
p
αPV mAb-2nd-A488
500 µm
q
αPV mAb-FL550
500 µm

Sup. Figure 15. Validation of immunofluorescence immunohistochemistry by scFv probes and their parental mAbs (part 3).

Cerebellum Crus 1 sections were immunolabeled with a calbindin-specific scFv (**a**), or its parental mAb and secondary antibody conjugated with Alexa Fluor 488 (**b**), the mAb conjugated with FL550 (**c**), or a commercial calbindin-specific pAb and secondary $(Fab)_2$ conjugated with Alexa Fluor 594 (**d**). **e**, Schematics showing the cutting orientation that is parallel to the lobule of Crus 1, which intersects perpendicular to the planer Purkinje cells in Crus 1. **f**, Sections cut in this orientation immunolabeled with the mAb conjugated with FL550. The boxed inset is shown enlarged in the adjacent panel. Whole-section images of cerebellum Crus 1 sections immunolabeled with a calbindin-specific scFv (**g**), or its parental mAb and secondary antibody conjugated with Alexa Fluor 488 (**h**), or the mAb conjugated with FL550 (**i**). Arrows indicate labeled cell nuclei of Purkinje cells. Arrowheads indicate the labeled axons. n=3 experiments for **a**-**b**; CB, calbindin; n=2 experiments for **c**-**d**, **f-i**.

Cerebellum Crus 1 sections were immunolabeled with a parvalbumin-specific scFv (**j**), or its parental mAb and secondary antibody conjugated with Alexa Fluor 488 (**k**), the mAb conjugated with FL550 (**l**), or a commercial parvalbumin-specific pAb and secondary $(Fab)_2$ conjugated with Alexa Fluor 594 (**m**). **n**, Sections cut in this orientation in **e** immunolabeled with the mAb conjugated with FL550. The boxed inset is shown enlarged in the adjacent panel. Whole-section images of cerebellum Crus 1 sections immunolabeled with a parvalbumin-specific scFv (**o**), or its parental mAb and secondary antibody conjugated with Alexa Fluor 488 (**p**), or the mAb conjugated with FL550 (**q**). Arrows indicate labeled cell nuclei of Purkinje cells. Arrowheads indicate the labeled axons; PV, parvalbumin; n=3 experiments for **j**-**k**; n=2 experiments for **l**-**q**.

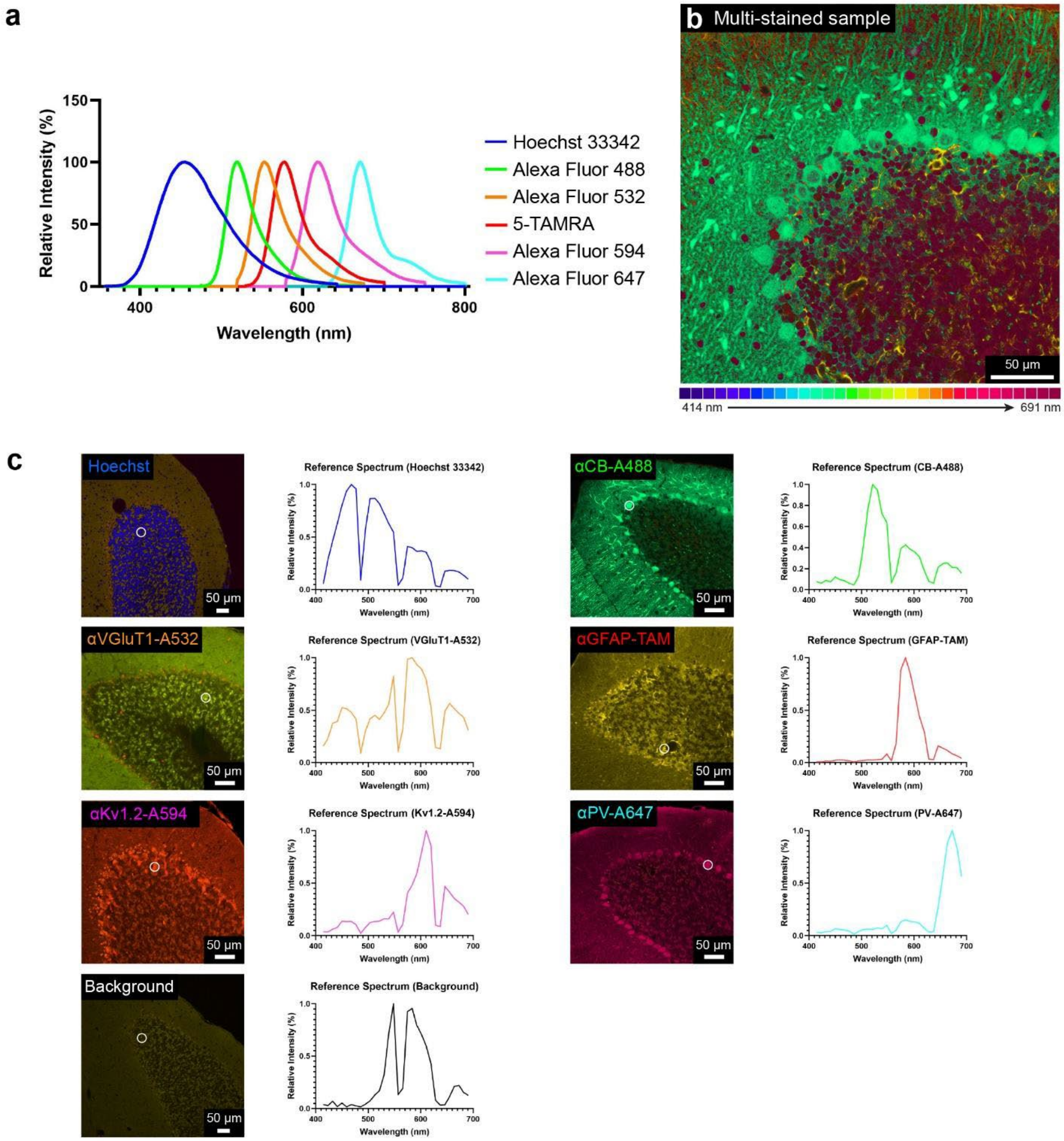

Sup. Figure 16. Technical details of linear unmixing.

a, The excitation and emission spectra of the six fluorescent dyes used in the multi-scFv labeling. **b**, An image slice from the lambda stack of the multicolor sample with a depth of 52 µm. The 32 channels (from 414 nm to 691 nm) in the lambda mode were labeled with different colors. **c**, the lambda stacks acquired from the individually labeled samples. The reference spectrum for each dye was extracted from the pixels labeled by the white circle in each lambda stack image.

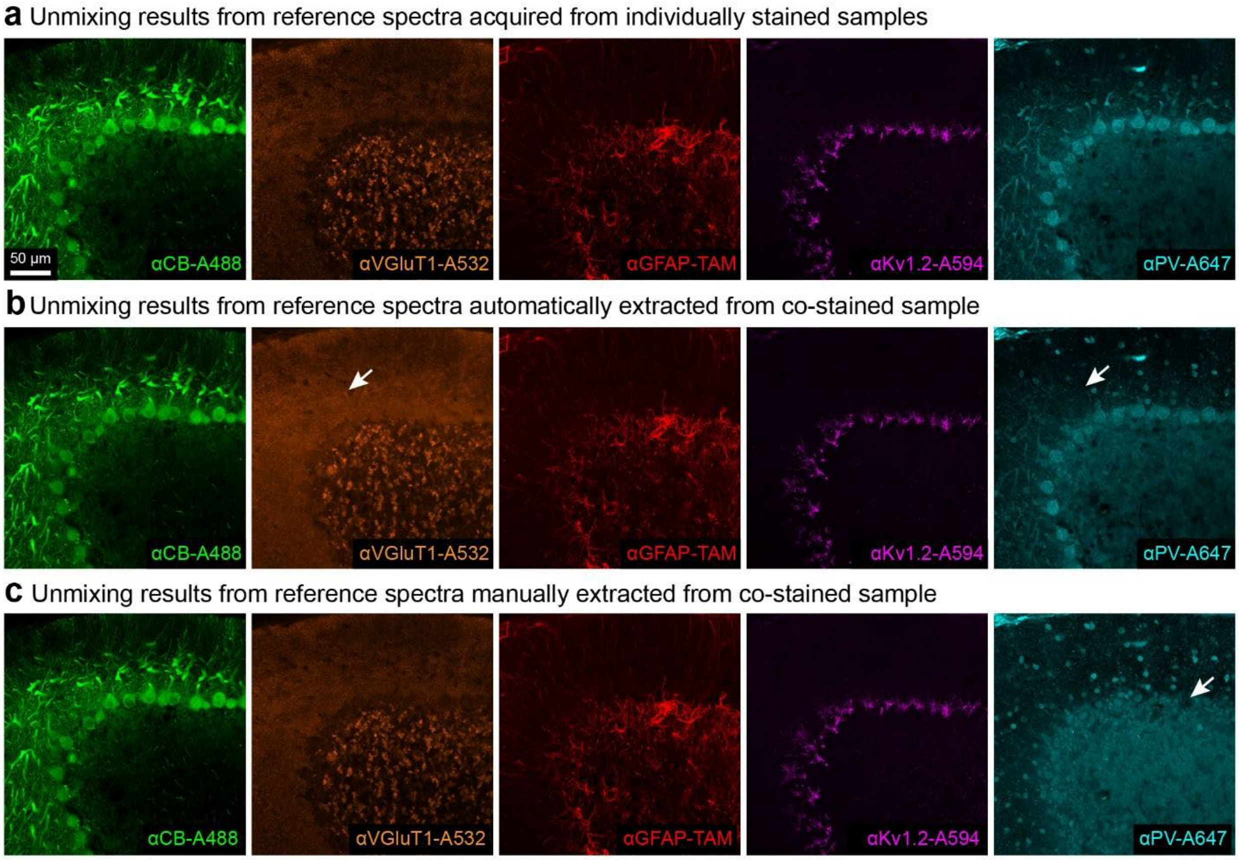

Sup. Figure 17. Comparison of linear unmixing results using reference spectra extracted in three different ways.

White arrows in **b** indicate the fluorescence signals that should be in the Alexa Fluor 647 channel, which were separated into the Alexa Fluor 532 channel instead. White arrows in **c** indicate where the fluorescence signals from the Purkinje cell bodies were missing in the Alexa Fluor 647 channel.

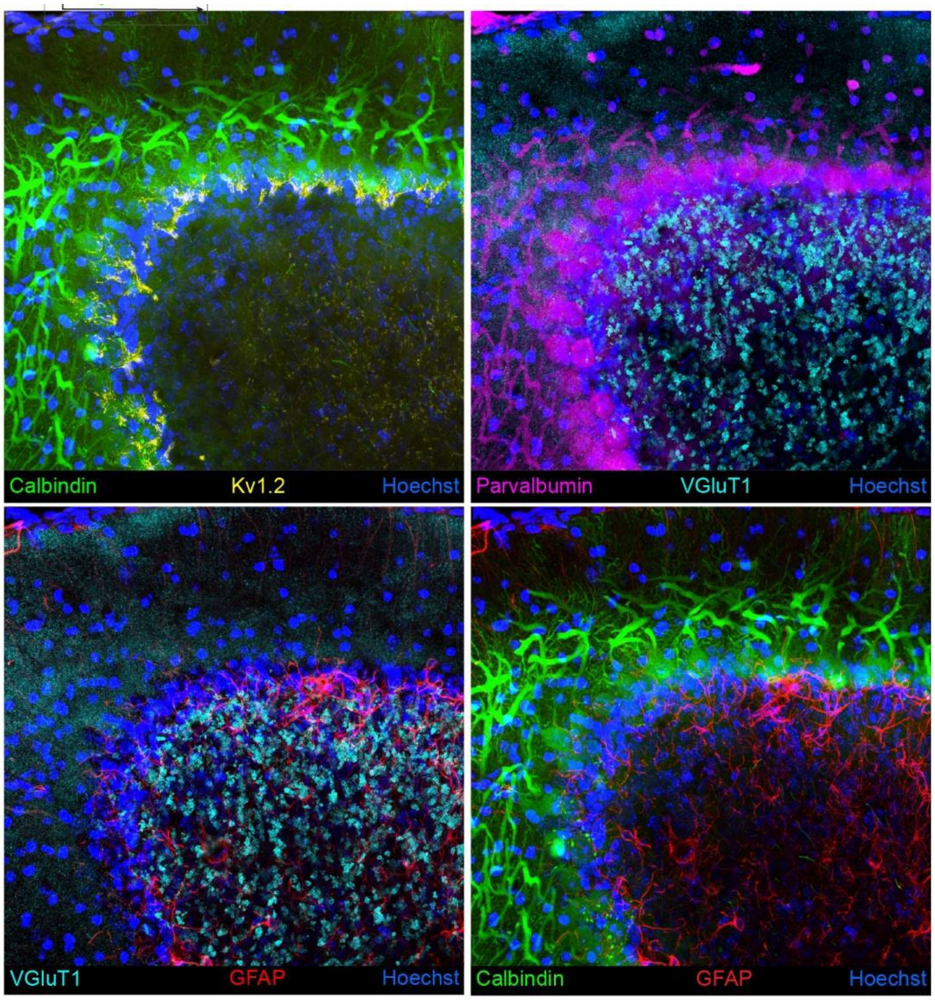

Sup. Figure 18. Three-channel maximum intensity projection images of the multi-color confocal fluorescence image stack in Figure 2 c.

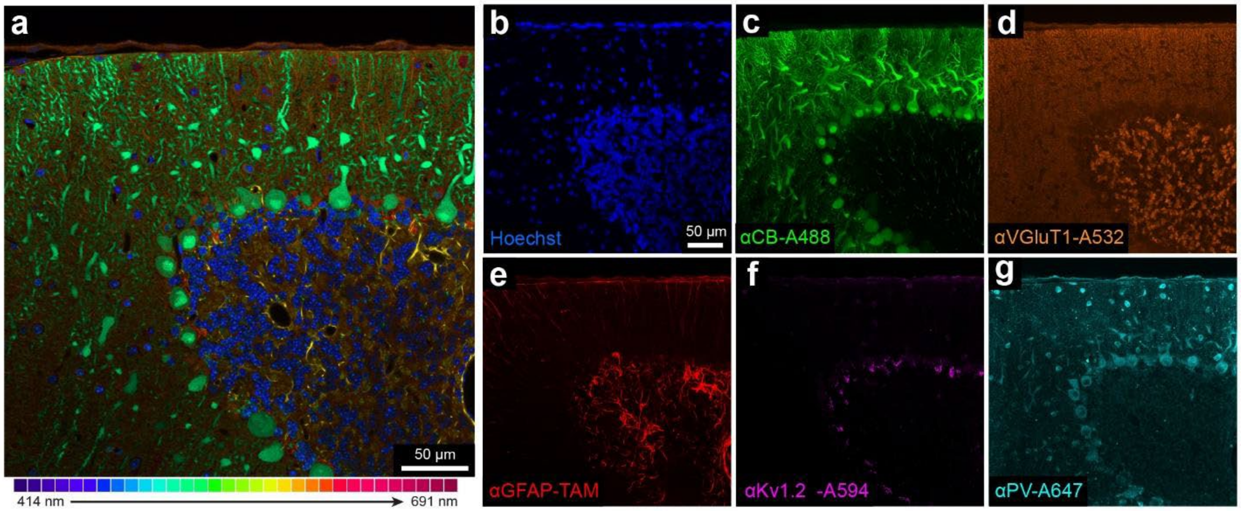

Sup. Figure 19. The results of combining the Hoechst channel into linear unmixing of confocal micrographs (n=1 experiment).

a, An image slice from the lambda stack of the multicolor sample with a depth of 49 µm. The 32 channels (from 414 nm to 691 nm) in the lambda mode were labeled with different colors. **b**-**g**, the linear unmixing results of the six channels (Hoechst, Alexa Fluor 488, Alexa Fluor 532, 5-TAMRA, Alexa Fluor 594, Alexa Fluor 647).

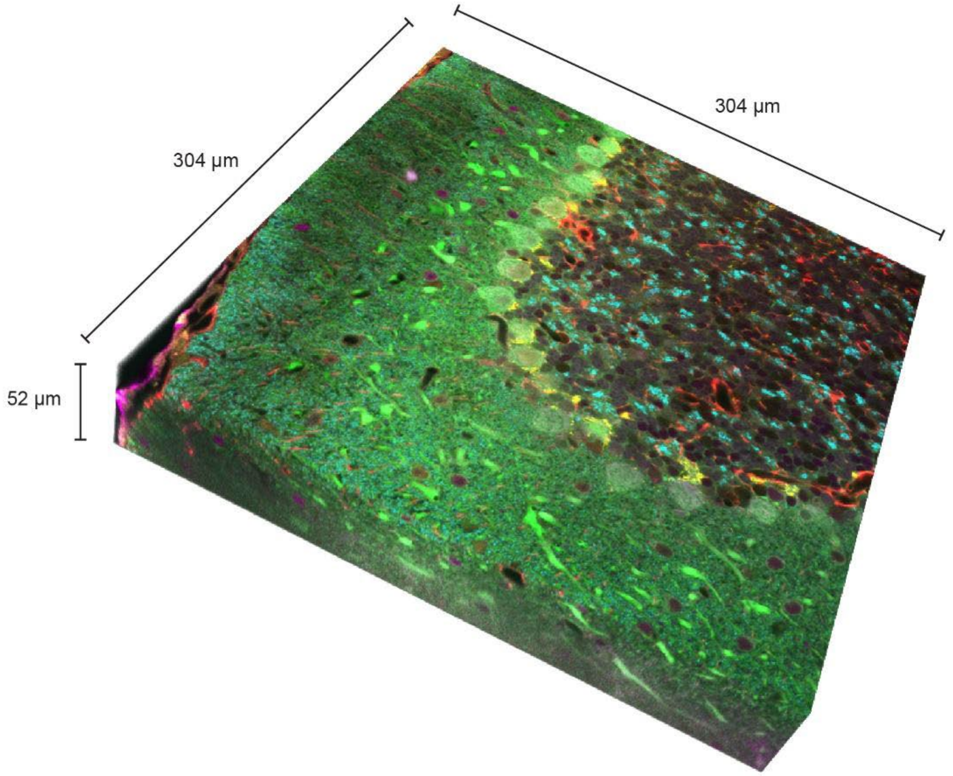

Sup. Figure 20. The dimensions of the multi-color confocal fluorescence image volume acquired by scFv-enabled immunofluorescence and linear unmixing (n=1 experiment).

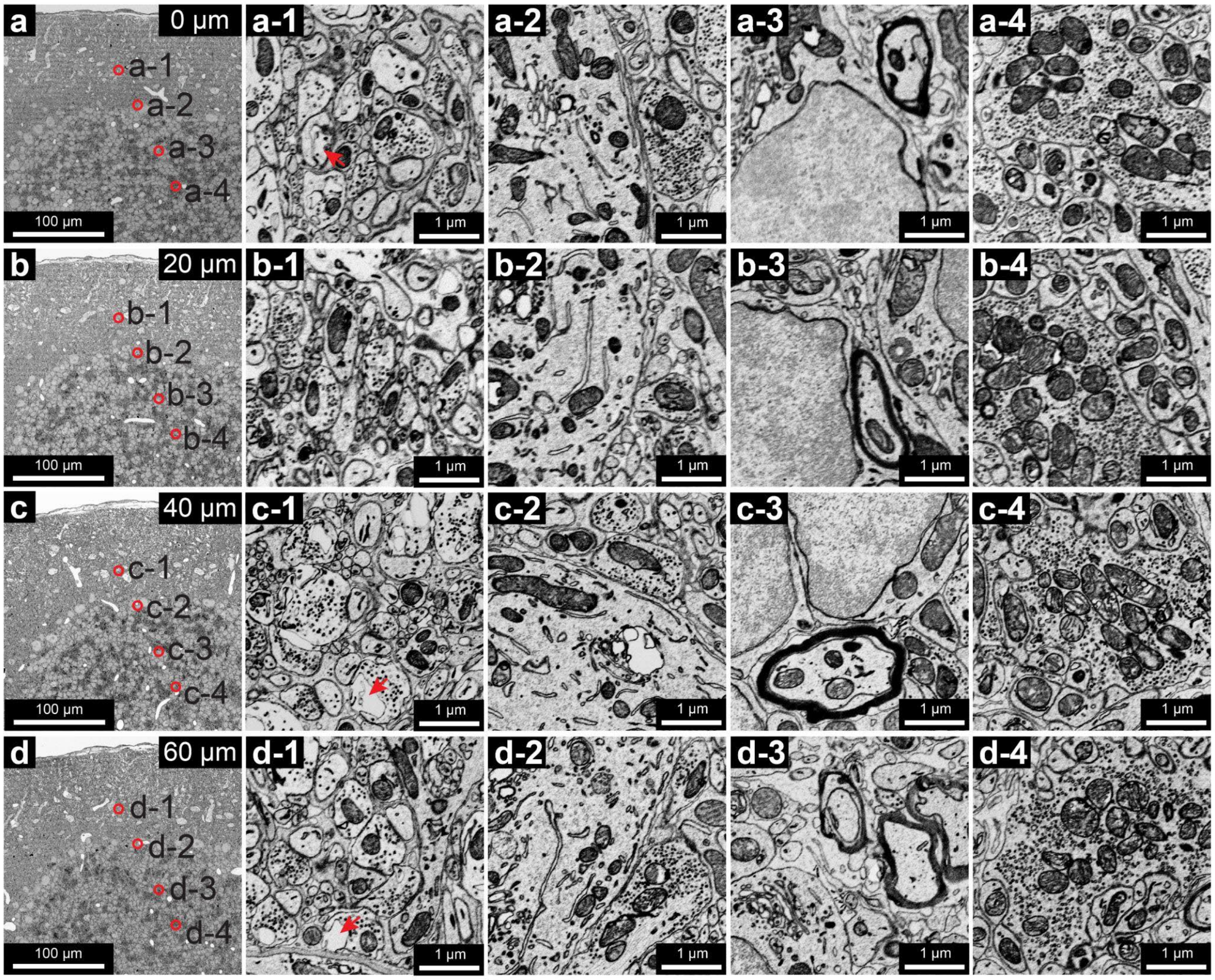

Sup. Figure 21. Well-preserved ultrastructure from the surface (a) to the middle (d) of the 120-µm section (n=2 experiments).

Panels **1-4** in **a-d** show the ultrastructure at the locations labeled by the red circles in the right panels. 0, 20, 40, 60-µm in each panel means the distance of the ultrathin section from the surface of the tissue section. Arrows indicate the artifacts potentially caused by prolonged incubation with scFvs for immunolabeling.

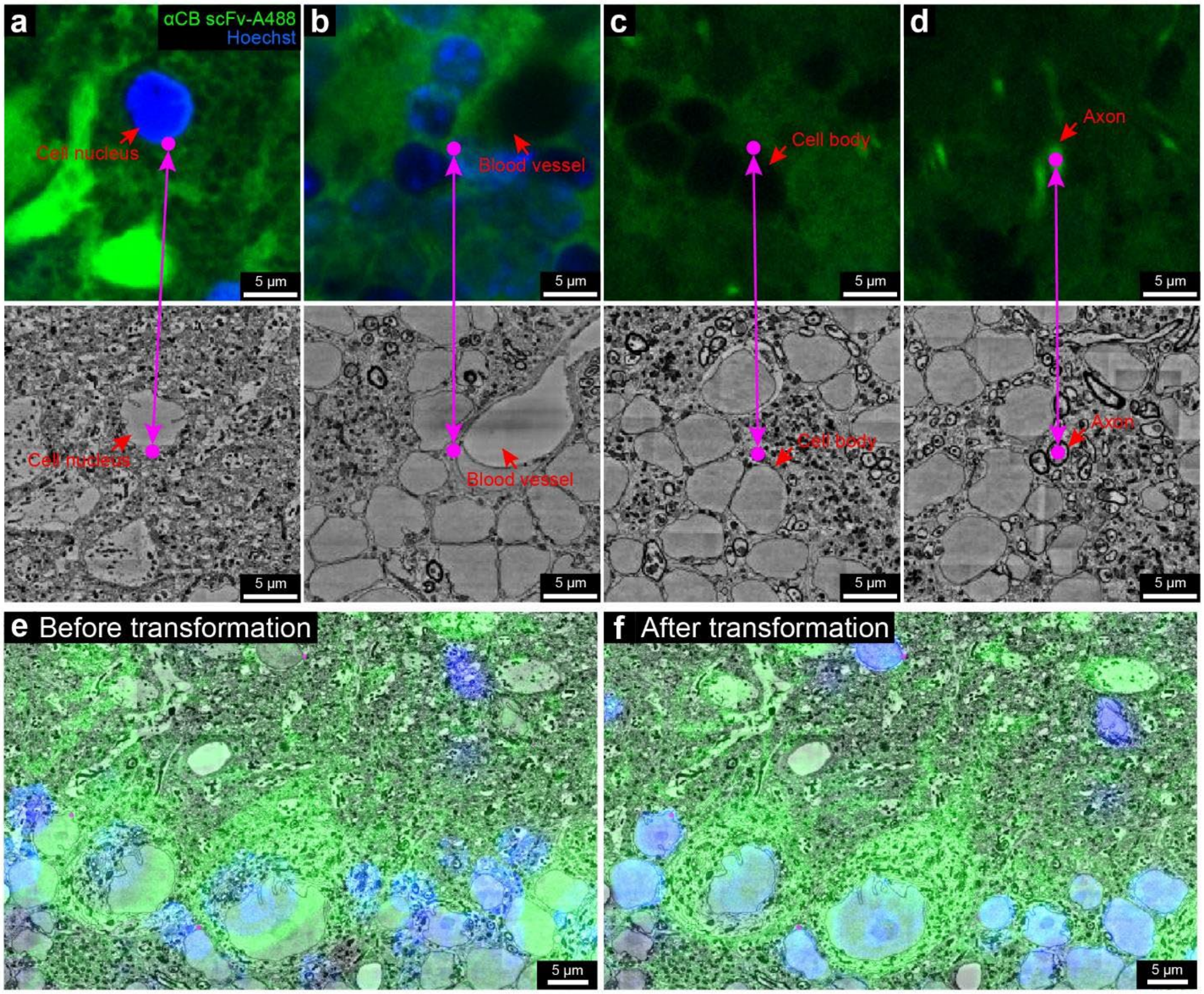

Sup. Figure 22. Co-registration between fluorescence image volume and the high-resolution EM volume (n=1 experiment).

a-**d,** landmark points that corresponded to the same sites in the two volumes were placed on blood vessels, cell nuclei, cell bodies, and axons. **e**-**f** show the overlays between the fluorescence image and the EM image before and after the transformation of the fluorescence image volume was performed based on the point correspondences.

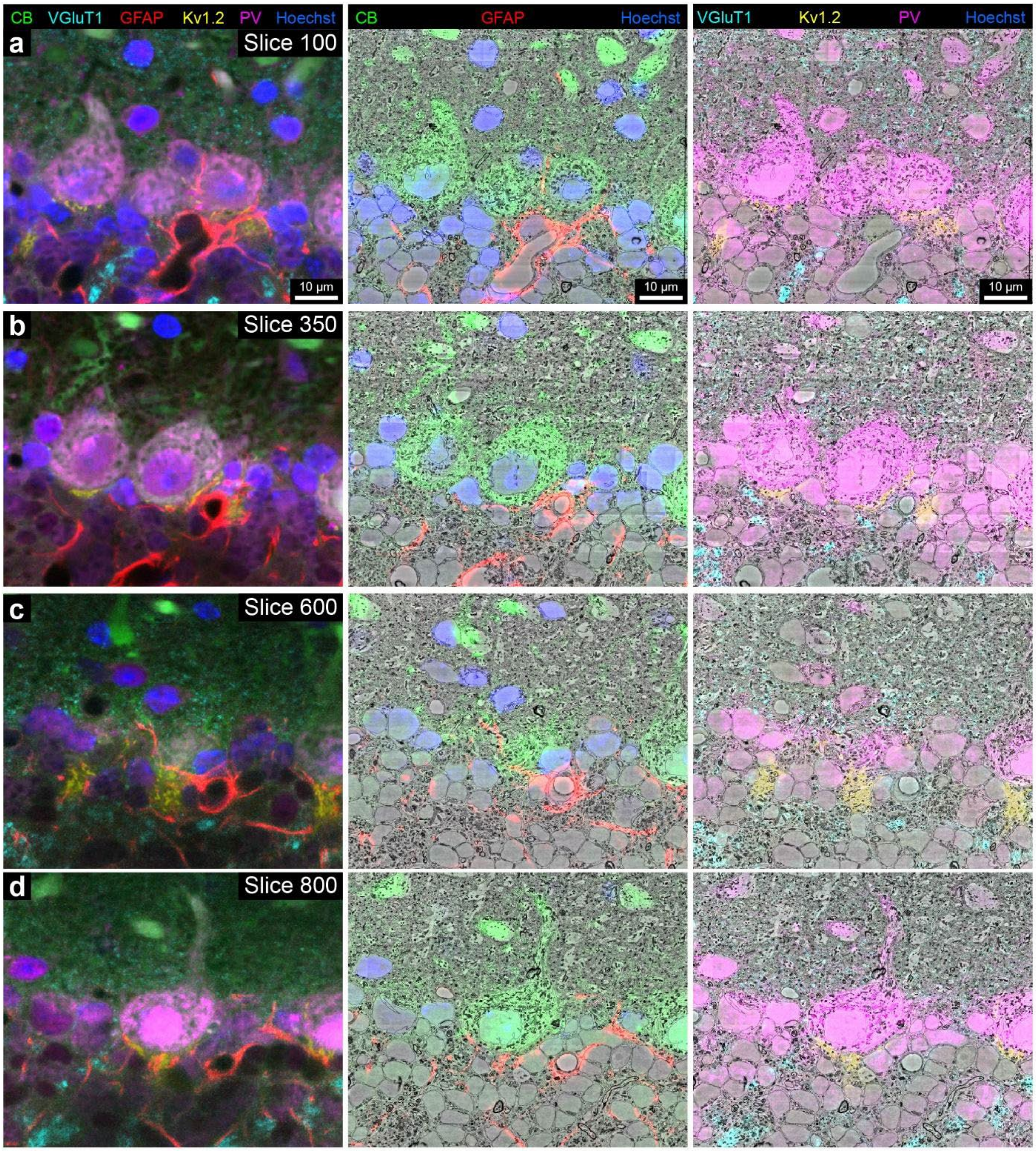

Sup. Figure 23. Demonstration of the overlay between fluorescence signals and EM ultrastructure throughout the vCLEM dataset (n=848 slices).

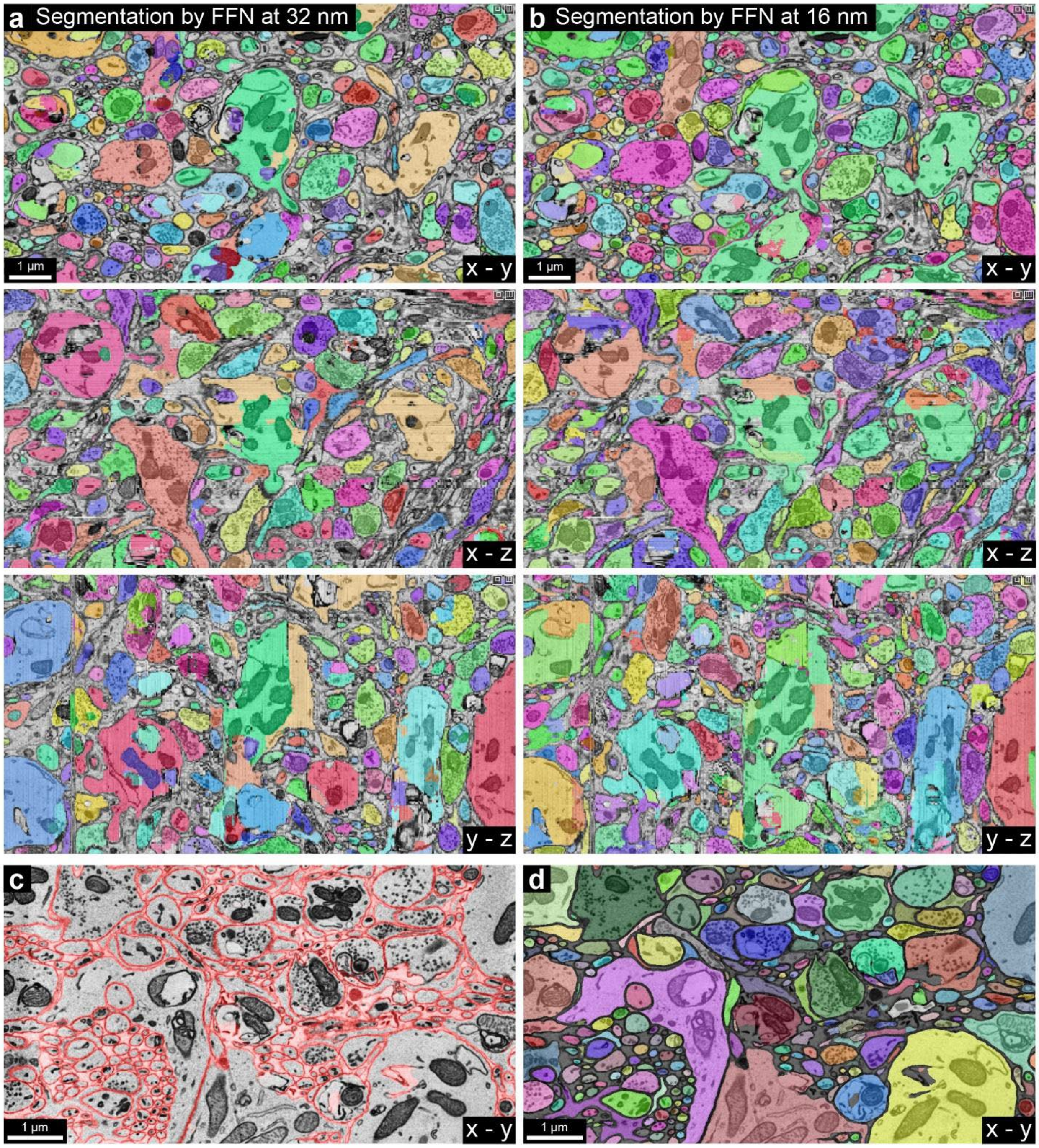

Sup. Figure 24. Automatic segmentation results. (n=848 slices)

3D segmentation results at 32 nm (**a**) and at 16 nm (**b**) by FFN [2]. Membrane prediction results at 8 nm (**c**) and 2D segmentation results at 8 nm (**d**) by Cross-Classification Clustering [3].

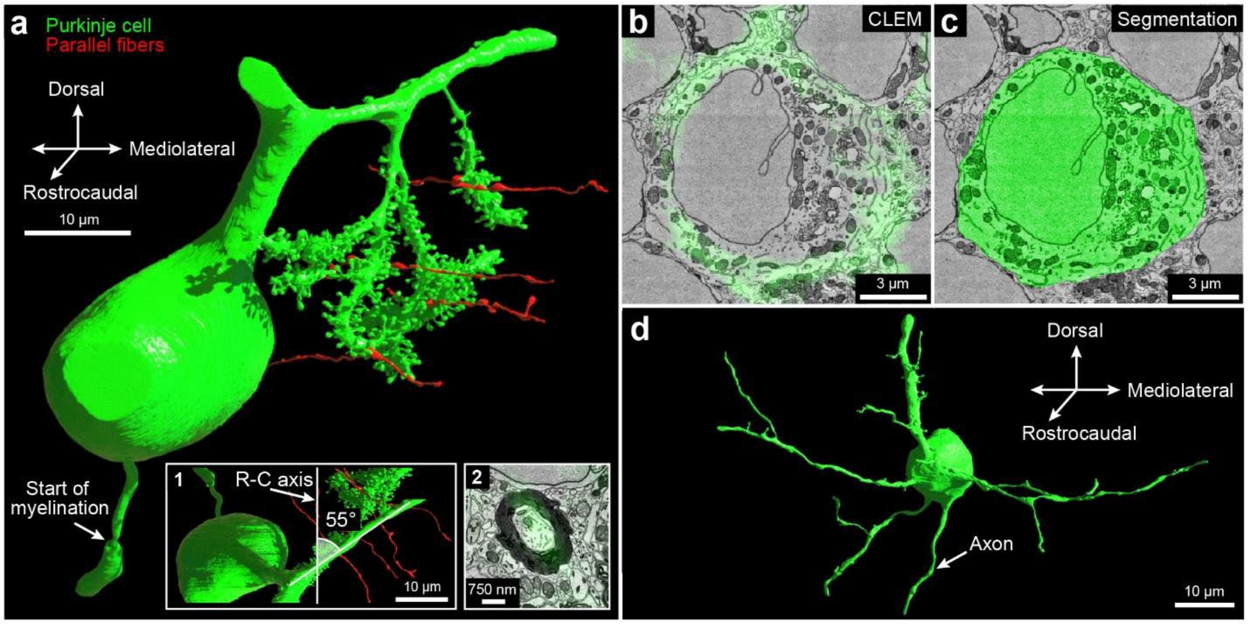

Sup. Figure 25. 3D reconstruction of cells labeled by the calbindin-specific scFv probe.

a, 3D reconstruction of the Purkinje cell (n=1) labeled in Figure 4 a (green) and four parallel fibers (red) that made synapses on the Purkinje cell. Inset **1**, the reconstructed Purkinje cell is viewed in the rostrocaudal-mediolateral plane. The dendritic tree of this Purkinje cell was not perpendicular to the rostral-caudal axis but intersected at the axis at an angle of around 55°. Inset **2**, the 2D CLEM image showing the fluorescence signal (green) of the calbindin-specific scFv probe overlaps with a heavily myelinated axon. **b**, the 2D CLEM image showing the fluorescence signal (green) of the calbindin-specific scFv probe overlaps with a Golgi cell (n=1). **c**, EM image showing the 2D segmentation (green) of the calbindin-positive Golgi cell based on staining shown in **b** (n=1). **d**, 3D reconstruction of the Golgi cell labeled in **b** (n=1).

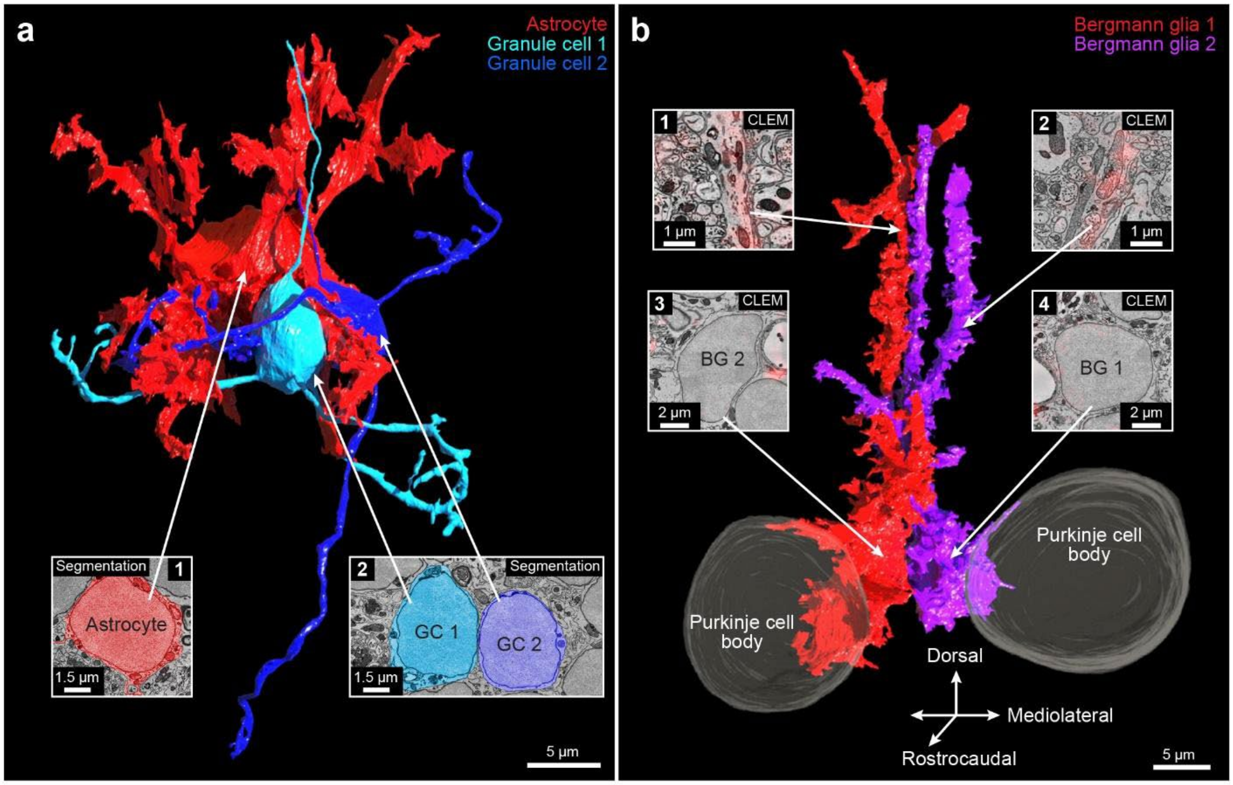

Sup. Figure 26. 3D reconstruction of cells labeled by the GFAP-specific scFv probe.

a, 3D reconstruction of the astrocyte labeled in Figure 4 e (red) (n=1) and two nearby granule cells (cyan and blue) (n=2). Insets, EM image showing the 2D segmentations of the cell bodies of the reconstructed astrocyte and granule cells. **b**, 3D reconstruction of the two labeled Bergmann glia labeled in Figure 4 k (n=2) (red) and two nearby Purkinje cells (n=2). Insets **1** and **2**, the 2D CLEM images showing the fluorescence signal (red) of the GFAP-specific scFv probe overlap with the processes of the Bergmann glia. In insets **3** and **4**, the 2D CLEM images showing the fluorescence signal (red) of the GFAP-specific scFv probe do not overlap with the cell bodies of the Bergmann glia.

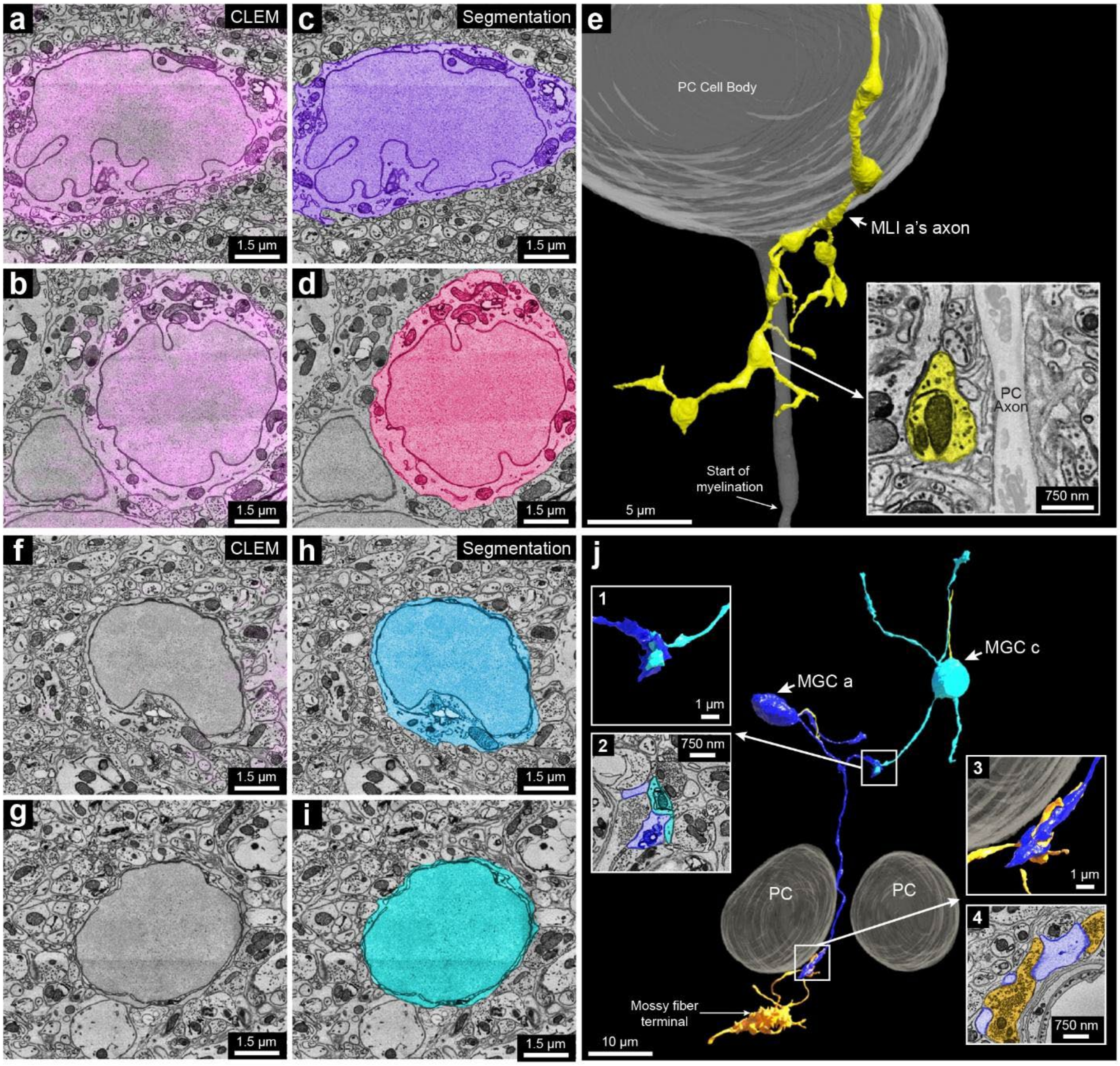

Sup. Figure 27. 3D reconstruction of the molecular layer interneurons and granule cells.

a-**b**, Representative 2D CLEM image (n=22) showing the fluorescence signals (magenta) of the parvalbumin-specific scFv probe overlap cell bodies of MLI b and c. **b**-**d**, EM image showing the 2D segmentation (purple and magenta) of MLI b and (n=2). **e**, the axon of MLI a was part of the pinceau structure that surrounds a Purkinje cell's axon initial segment (inset) (n=1). **f**-**g**, Representative 2D CLEM image (n=7) showing the fluorescence signals (magenta) of the parvalbumin-specific scFv probe do not overlap cell bodies of MGC b and c. **h**-**i**, EM image showing the 2D segmentation (blue and cyan) of MGC b and c (n=2). **j**, 3D reconstruction MGC a and MGC c (n=2). These two cells formed a glomerulus (insets **1** and **2**) (n=1). MGC a received synaptic input from a mossy fiber terminal (insets **3** and **4**) (n=1).

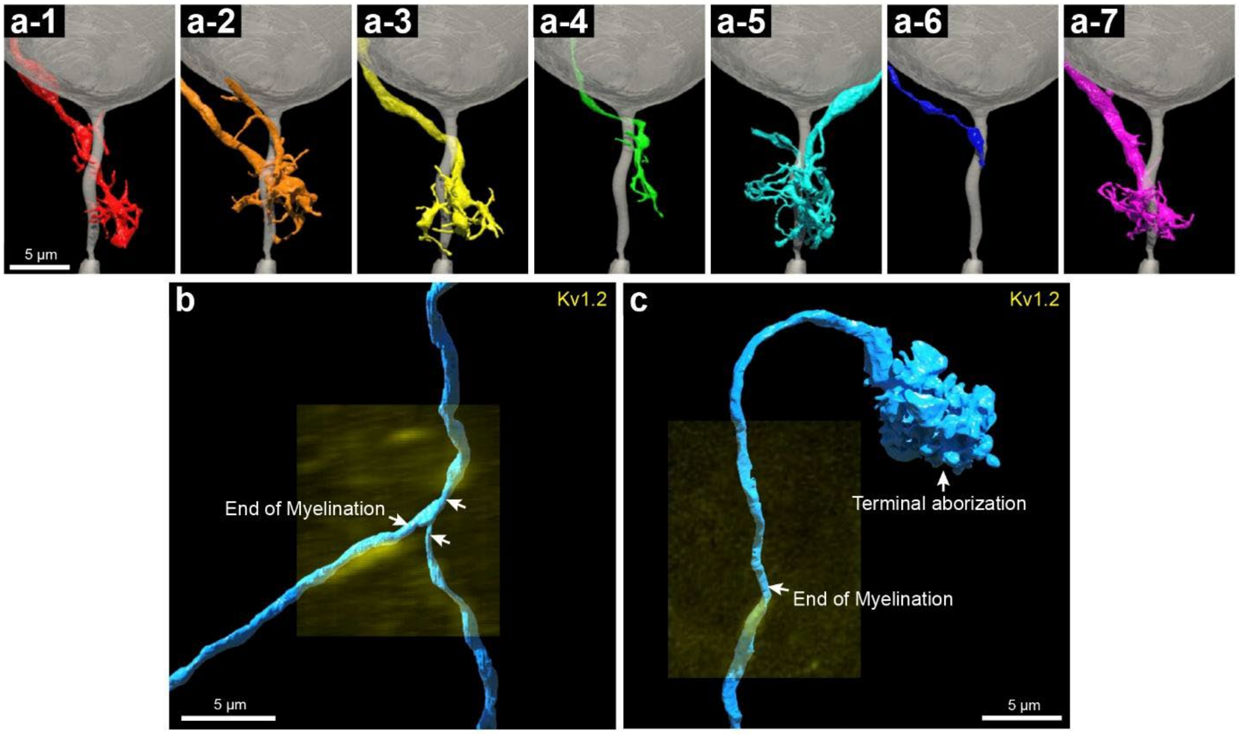

Sup. Figure 28. 3D reconstruction of axonal terminals in a pinceau and the mossy fibers labeled with the Kv1.2-specific scFv.

a-1 to **a-7**, seven individual axon terminals (n=7) labeled by the Kv1.2-specific scFv probe at the pinceau structure in Figure 6 a-c. **b**, Representative 3D reconstruction of an axon with Kv1.2 positive juxtaparanodal labeling (yellow fluorescence) at a branching point (n=2). **c**, Representative 3D reconstruction of an axon with Kv1.2 positive juxtaparanodal labeling (yellow fluorescence) at the site where the myelination ended (n=5). This axon ended in a terminal axonal arborization (arrow) in the granule cell layer.

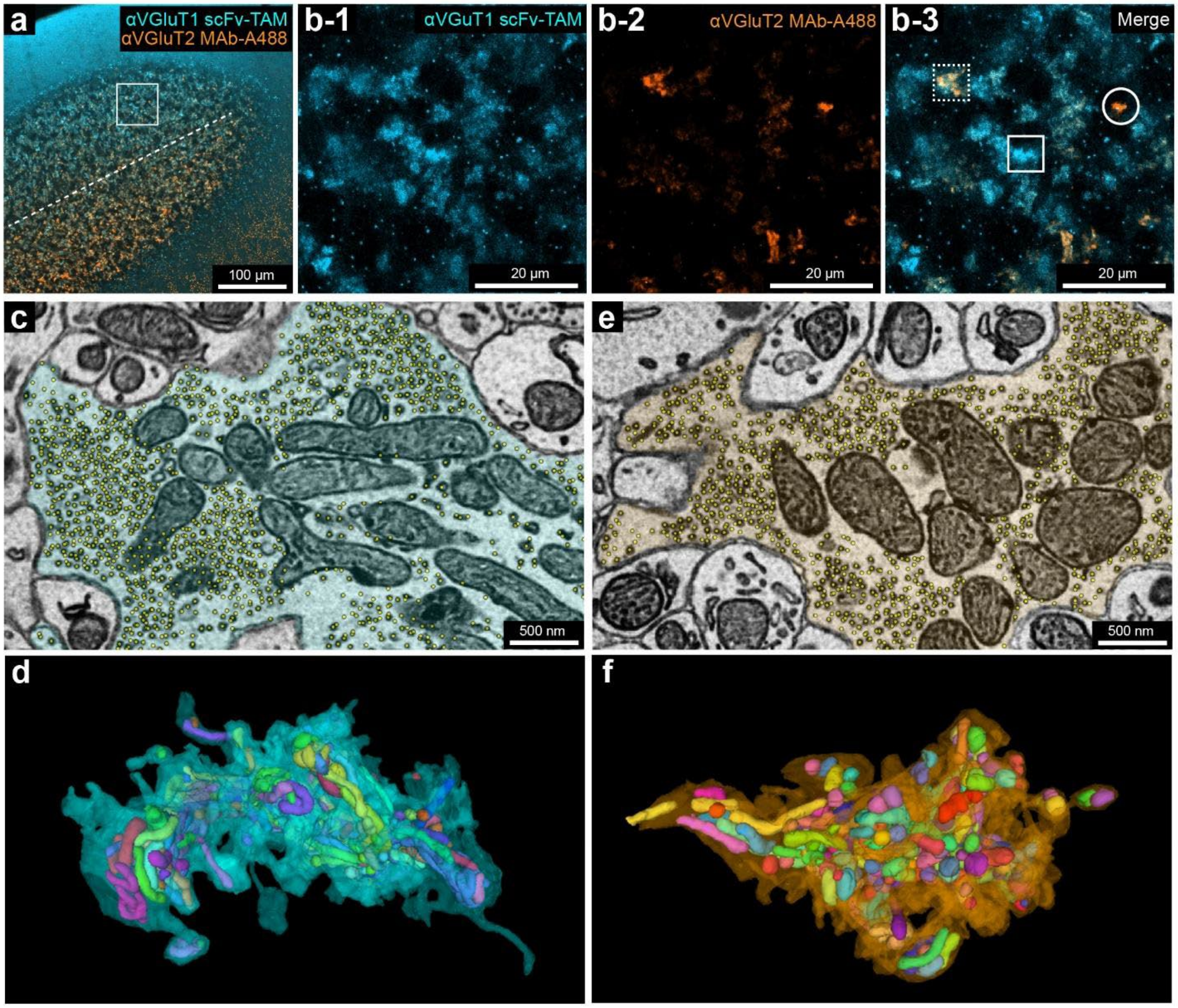

Sup. Figure 29. Three types of mossy fiber terminals identified by double immunofluorescence of VGluT1/2 and synaptic vesicle and mitochondria detection results.

a, Representative confocal image (n=3 experiments) of a section from the cerebellum Crus 1 stained with a VGluT1-specific scFv probe conjugated with Alexa Fluor 5-TAMRA and a VGluT2-specific scFv probe with secondary antibodies conjugated with Alexa Fluor 488. The image was pseudo-colored. Note the labeling of VGLUT1 was stronger than the labeling of VGluT2 on the left side of the dotted line and vice versa. **b-1** to **b-3**, Single-channel and two-channel images enlarged from the boxed inset in **a**. The box with solid lines indicates a VGluT1 positive terminal. The box with dotted lines indicates a VGluT1/2 double positive terminal. The circle indicates a VGluT2 positive terminal. **c**-**d,** Representative synaptic vesicle and mitochondria detection results from a reconstructed VGluT1 positive terminal (n=10). Colored objects inside the transparent terminal are the detected mitochondria. **e**-**f,** Representative synaptic vesicle and mitochondria detection results from a reconstructed VGluT1 negative terminal (n=10). Colored objects inside the transparent terminal are the detected mitochondria.

Sup. Table 1. The final concentrations of scFv and nanobody probes used in the immunofluorescence of this work.

Target	Clone No.	Fluorescent dyes conjugated	Final conc.
**GFP (scFv)**	N86/38	5-TAMRA	0.01 mg/ml
**GFP (nanobody)**	Enh [4]	Alexa Fluor 647	0.01 mg/ml
**Calbindin**	L109/57	5-TAMRA, Alexa Fluor 488	0.01 mg/ml
**GFAP**	N206B/9	5-TAMRA	0.01 mg/ml
**VGluT1**	N28/9	5-TAMRA, Alexa Fluor 532	0.015 mg/ml
**PSD-95**	K28/43	5-TAMRA	0.01 mg/ml
**Kv1.2**	K14/16	Alexa Fluor 594	0.02 mg/ml
**Parvalbumin**	L114/81	Alexa Fluor 647	0.01 mg/ml
**Neuropeptide Y**	L115/13	Alexa Fluor 594	0.01 mg/ml

Sup. Table 2.The dilution ratios/final concentrations of primary antibodies used in the immunofluorescence of used in this work.

Target	Clone No.	Vendor	Catalog No.	Dilution Ratio/Final concentration
**GFP**	N.A.	ThermoFisher	A-31852	1:200
**Calbindin**	L109/57	Antibodies Incorporated	75-448	1:200
**Calbindin**	L109/39	NeuroMab	RRID: AB_2877181	22.5 μg/ml
**GFAP**	N206B/9	Antibodies Incorporated	75-279	1:200
**GFAP**	N206B/8	NeuroMab	RRID: AB_2877358	11.1 μg/ml
**GFAP**	N206B/9	NeuroMab	N.A.	1:5
**VGluT1**	N28/9	Antibodies	75-066	1:200

		Incorporated		
**VGluT2**	N28/29	Antibodies Incorporated	75-067	1:200
**PSD-95**	K28/43	Antibodies Incorporated	75-028	1:200
**Kv1.2**	K14/16	Antibodies Incorporated	75-008	1:500
**Kv2.1**	N.A.	In-house polyclonal rabbit antibody [1]	N.A.	1:20
**Parvalbumin**	L114/81	Antibodies Incorporated	75-479	1:200
**Neuropeptide Y**	L115/13	Antibodies Incorporated	75-456	1:200
**Calbindin**	L109/57	Antibodies Incorporated	75-448-FL550	1:100
**Calbindin**	L109/57	NeuroMab	N.A.	3 μg/ml
**Calbindin**	N.A.	Synaptic System	214 005	1:100
**Parvalbumin**	L114/81	Antibodies Incorporated	75-479-FL550	1:100
**Parvalbumin**	L114/81 R (IgG2a)	NeuroMab; [5]	N.A.	1:2
**Parvalbumin**	N.A.	Abcam	ab11427	1:250
**FLAG**	N.A.	Millipore	F7425	2.5 μg/ml
**Pan-QKI**	N147/6	NeuroMab	N.A.	2.5 μg/ml
**GAD1**	L127/8	NeuroMab	N.A.	5 μg/ml

Sup. Table 3. The dilution ratios/final concentrations of secondary antibodies used in the immunofluorescence of used in this work.

Name	Fluorescence dye conjugated	Vendor	Catalog No.	Dilution Ratio
**Goat anti-Mouse IgG1 Secondary Antibody**	Alexa Fluor 488	ThermoFisher	A-21121	1:100
**Goat anti-Mouse IgG2a Secondary Antibody**	Alexa Fluor 488	ThermoFisher	A-21131	1:100; 2 μg/ml
**Goat anti-Mouse IgG2b Secondary Antibody**	Alexa Fluor 488	ThermoFisher	A-21141	1:100; 2 μg/ml
**AffiniPure™ Goat Anti-Guinea Pig IgG, F(ab')$_2$ fragment specific**	Alexa Fluor 594	Jackson Immuno Research	106-585-006	1:100
**AffiniPure™ Goat Anti-Rabbit IgG, F(ab')$_2$ fragment specific**	Alexa Fluor 594	Jackson Immuno Research	111-585-006	1:100
**Goat anti-Mouse IgG1 Secondary Antibody**	CF770	Biotium	20254-1	1:2500
**Donkey anti-Rabbit IgG (H+L) Secondary Antibody**	CF750	Biotium	20298-1	2 μg/ml
**Goat anti-Mouse IgG2b Secondary Antibody**	Alexa Fluor 594	ThermoFisher	A-21145	1:2500
**Goat anti-Rabbit IgG (H+L) Secondary Antibody**	Alexa Fluor 488	ThermoFisher	A-11008	1:2500
**Goat anti-Rabbit IgG (H+L) Secondary Antibody**	Alexa Fluor 647	ThermoFisher	A-21244	2 μg/ml
**Goat anti-Mouse IgG1 Secondary Antibody**	Alexa Fluor 555	ThermoFisher	A-21127	2 μg/ml

**Goat anti-Mouse IgG2b Secondary Antibody**	Alexa Fluor 555	ThermoFisher	A-21147	2 µg/ml
**Goat anti-Mouse IgG1 Secondary Antibody**	Alexa Fluor 647	ThermoFisher	A-21240	2 µg/ml
**Goat anti-Mouse IgG2b Secondary Antibody**	Alexa Fluor 647	ThermoFisher	A-21242	2 µg/ml

Sup. Table 4. Information on the validation of the scFvs and their parental mAbs.

**Target**	**Clone No.**	**mAb validation**				**scFv validation**
		**COS-IF**	**Brain IB**	**Brain IHC**	**KO Brain IHC**	**Method**
GFP	N86/38	Pass	NA	NA	NA	COS-IF
Calbindin	L109/57	Pass	Pass	Pass	ND	Brain IHC and COS-IF
GFAP R416WT	N206B/9	Pass	Pass	Pass	Pass	Brain IHC and COS-IF
VGluT1	N28/9	Pass	Pass	Pass	ND	Brain IHC and COS-IF
PSD-95	K28/43	Pass	Pass	Pass	Pass	Brain IHC and COS-IF
Kv1.2	K14/16	Pass	Pass	Pass	Pass	Brain IHC and COS-IF
Parvalbumin	L114/81	Pass	Pass	Pass	ND	Brain IHC and COS-IF
NPY	L115/13	Pass	Fail	Pass	ND	COS-IF

Sup. Table 5. Information on the VGluT1 positive terminals.

**Positive terminal No.**	**Volume / µm^3**	**Vesicle Number**	**Vesicle density per µm^3**	**Mito volume / µm^3**	**Mito volume ratio**
**1**	123.19	436042	3540	24.13	19.59%
**2**	109.43	426878	3901	22.04	20.14%
**3**	85.79	292704	3412	20.81	24.26%
**4**	124.89	409789	3281	30.94	24.77%
**5**	97.90	233785	2388	29.45	30.08%
**6**	89.89	255883	2847	22.09	24.57%
**7**	92.86	277102	2984	22.19	23.90%

**8**	84.47	246042	2913	24.43	28.92%
**9**	75.39	269515	3575	15.90	21.09%
**10**	109.52	293002	2675	31.37	28.64%

Sup. Table 6 Information on the VGluT1 negative terminals.

**Negative terminal No.**	**Volume / µm^3**	**Vesicle Number**	**Vesicle density per µm^3**	**Mito volume / µm^3**	**Mito volume ratio**
**1**	71.81	196695	2739	20.56	28.63%
**2**	92.23	268178	2812	24.62	26.70%
**3**	75.40	144275	1913	24.54	32.55%
**4**	33.73	97526	2891	10.56	31.31%
**5**	151.10	424845	2812	43.92	29.07%
**6**	61.82	142368	2303	17.92	29.00%
**7**	40.06	116529	2909	9.03	22.54%
**8**	68.55	213897	3120	16.70	24.36%
**9**	40.73	120864	2968	10.50	25.78%
**10**	42.79	141617	3309	9.37	21.90%

Supplementary References

1. Trimmer, J. S. Immunological identification and characterization of a delayed rectifier K+ channel polypeptide in rat brain. *Proc. Natl. Acad. Sci. U. S. A.* **88**, 10764–10768 (1991).
2. Januszewski, M. *et al.* High-precision automated reconstruction of neurons with flood-filling networks. *Nat. Methods* **15**, 605–610 (2018).
3. Meirovitch, Y. *et al.* Cross-classification clustering: An efficient multi-object tracking technique for 3-D instance segmentation in connectomics. *arXiv [cs.CV]* 8425–8435 (2018).
4. Kirchhofer, A. *et al.* Modulation of protein properties in living cells using nanobodies. *Nat. Struct. Mol.*

Biol. **17**, 133–138 (2010).

5. Andrews, N. P. *et al.* A toolbox of IgG subclass-switched recombinant monoclonal antibodies for enhanced multiplex immunolabeling of brain. *Elife* **8**, (2019).
